# Supplementary material for: Short- and long-read metagenomics of urban and rural South African gut microbiomes reveal a transitional composition and undescribed taxa
Source: Nat Commun. 2022 Feb 22;13:926. doi: 10.1038/s41467-021-27917-x (PMC8863827; doi:10.1038/s41467-021-27917-x)
Supplement: Supplementary file 1 — Supplementary Information [file 41467_2021_27917_MOESM1_ESM.pdf]

Supplementary Information

Table of Contents

Supplementary Figures....2
Supplementary Tables....36
Community engagement....41
Additional information on study sites....42
Microbiome and human genetic association testing....44
Supplementary References....46

Supplementary Figures

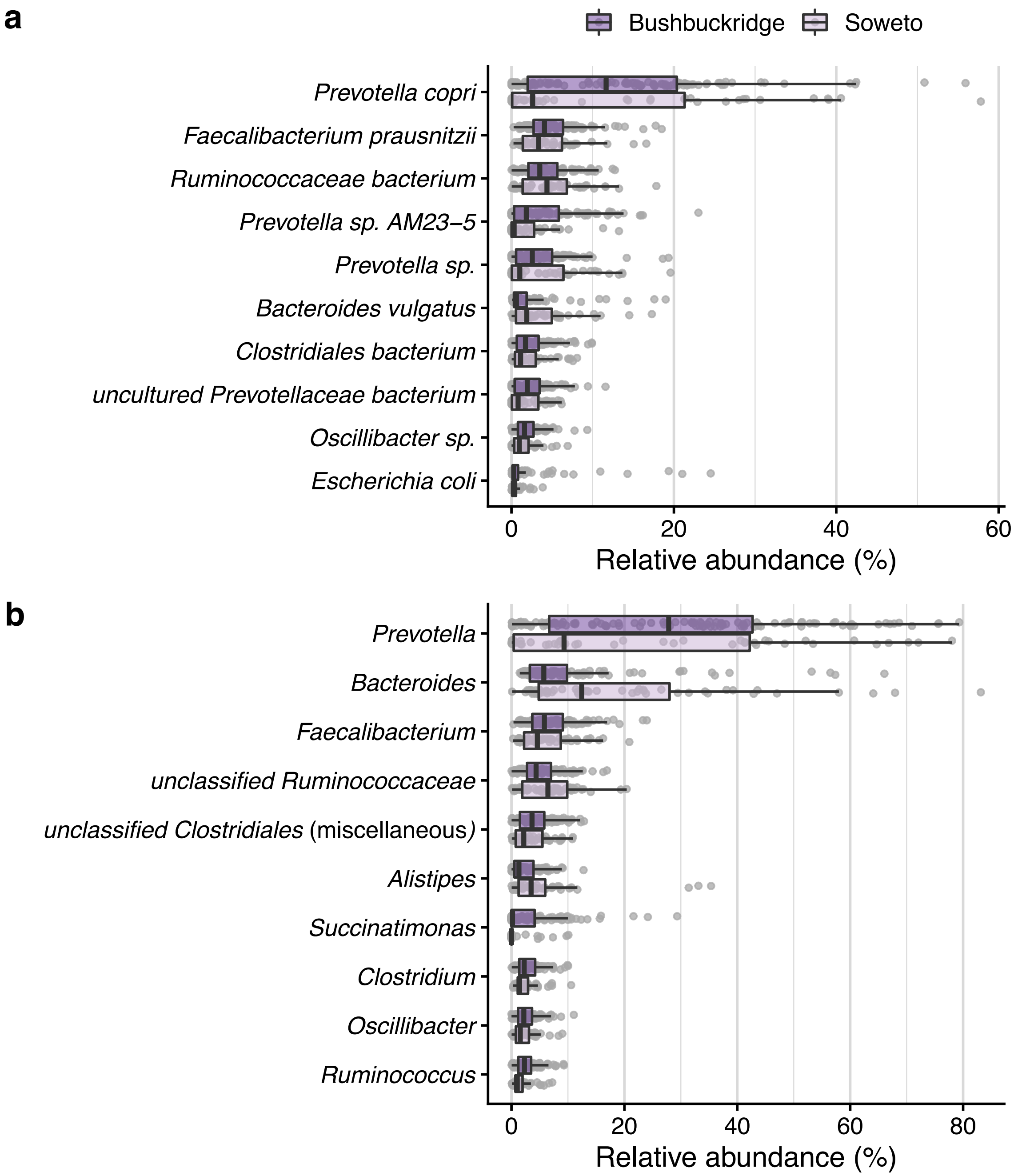

Supplementary Figure 1. Most abundant species and genera

Most abundant species (**a**) and genera (**b**) by mean relative abundance (total sum scaling) shown for samples from Bushbuckridge (n=118) and Soweto (n=51). Taxa are plotted in decreasing order of mean relative abundance calculated across both cohorts combined. Lower and upper box plot hinges correspond to the first and third quartiles,

upper and lower box plot whiskers represent the highest and lowest values within 1.5 times the interquartile range, and the vertical line represents the median.

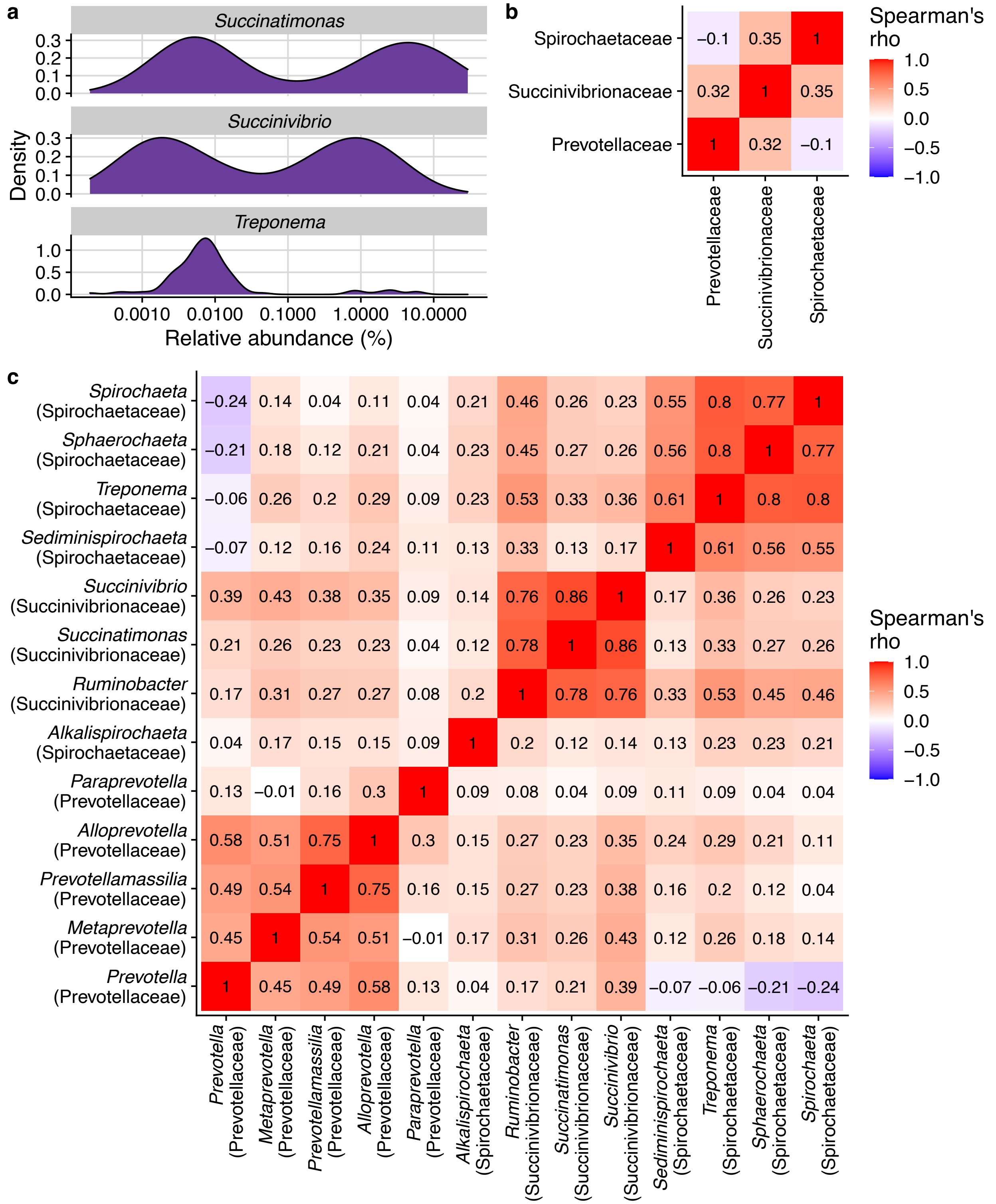

Supplementary Figure 2. Bimodal distribution of three VANISH taxa

a *Succinatimonas, Succinivibrio*, and *Treponema* relative abundance values follow a bimodal distribution in Bushbuckridge.

Across all South African samples, several VANISH families (**b**) and genera (**c**) are positively correlated, with the exception of *Prevotella* and genera of the family Spirochaetaceae, which are not correlated with *Prevotella* (*Treponema*) or weakly negatively correlated with *Prevotella* (*Spirochaeta*, *Sphaerochaeta*, *Sediminispirochaeta*).

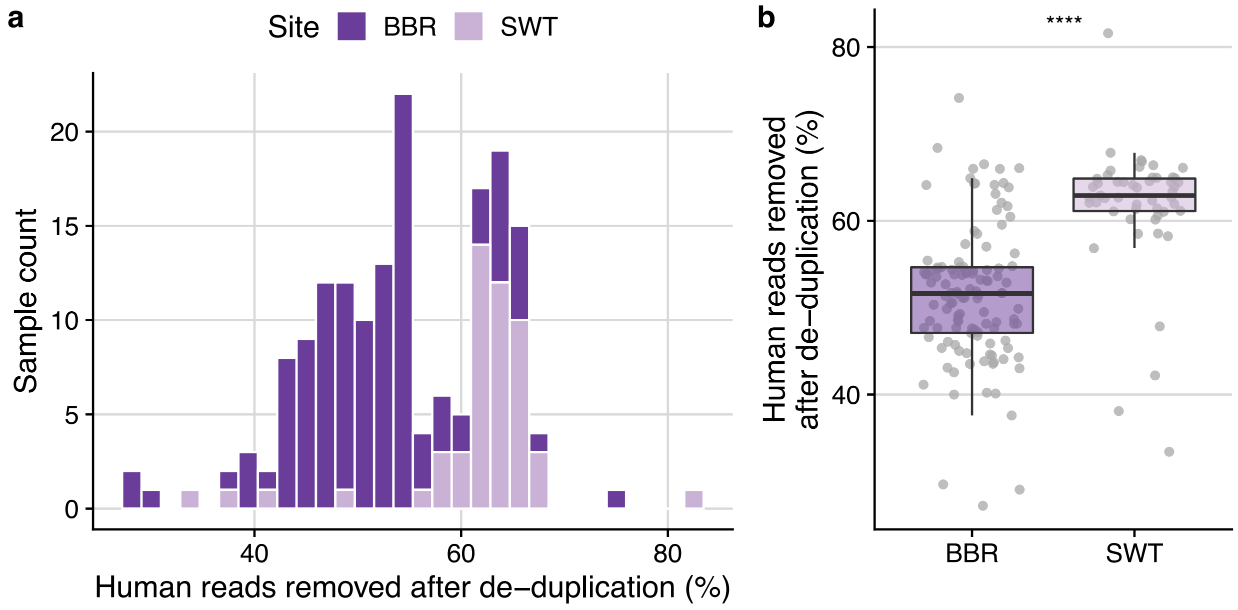

Supplementary Figure 3. Abundance of human reads in metagenomic sequencing Histogram (**a**) and box plots (**b**) indicating that the proportion of human reads removed after deduplication was found to be higher in the Soweto (SWT) cohort compared to Bushbuckridge (BBR) (two-sided Wilcoxon rank sum test, p = 1.661e-12; n=118 Bushbuckridge, n=51 Soweto). Significance values for Wilcoxon rank sum tests denoted as (****) for $p < 0.0001$. Lower and upper box plot hinges correspond to the first and third quartiles, upper and lower box plot whiskers represent the highest and lowest values within 1.5 times the interquartile range, and the horizontal line represents the median.

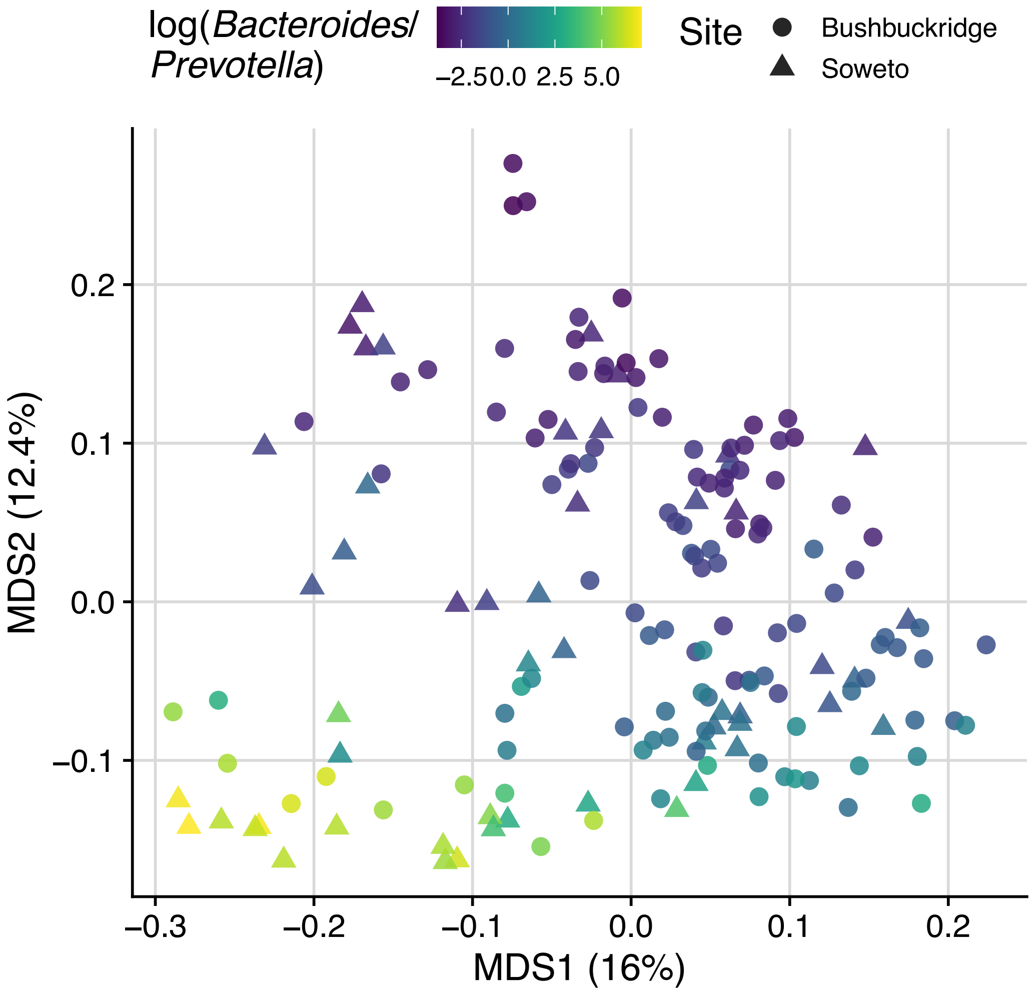

Supplementary Figure 4. Bacteroides/Prevotella gradient across study population Multidimensional scaling (MDS) of Bray-Curtis distance calculated from species classifications in South African microbiome samples (cumulative sum scaling normalized) colored by log ratio of the relative abundance of the genera *Bacteroides* and *Prevotella*. *Bacteroides* and *Prevotella* are major axes of variation across study samples.

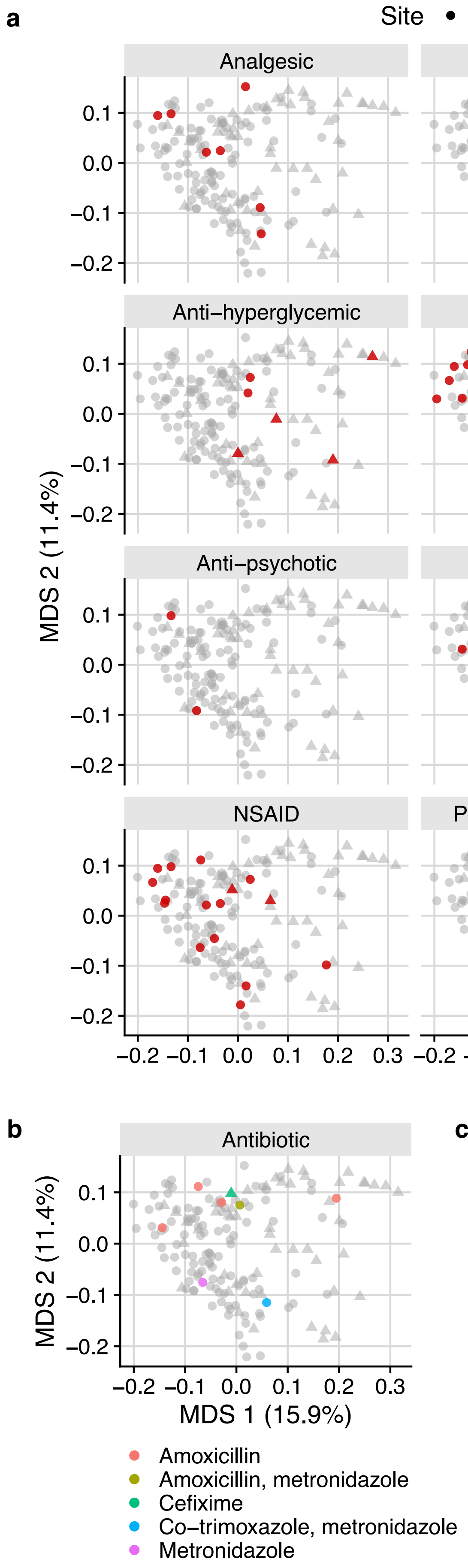

c

Category	R2	Pr(>F)	FDR
Analgesic	0.005	0.665	0.798
Antacid	0.006	0.370	0.798
Anti−diarrheal	0.005	0.471	0.798
Anti−hyperglycemic	0.010	0.040	0.240
Anti−hypertensive	0.005	0.807	0.807
Anti−parkinsonian	0.005	0.631	0.798
Anti−psychotic	0.005	0.646	0.798
Antibiotic	0.005	0.785	0.807
Diuretic	0.006	0.362	0.798
NSAID	0.007	0.243	0.798
Proton−pump−inhibitor	0.013	0.031	0.240
Statin	0.006	0.439	0.798

Supplementary Figure 5: Concomitant medications do not substantially impact community composition

Multidimensional scaling (MDS) of Bray-Curtis distance calculated from species classifications. Circles indicate participants from Bushbuckridge, triangles indicate participants from Soweto.

a Points are colored red if the participant was taking a medication of the corresponding class, patients not taking a medication of that class are shown in gray.

b Specific antibiotics taken by participants. Points are colored according to the antibiotic or combination of antibiotics reported.

c PERMANOVA R^2 values and nominal and adjusted p-values for the variation explained by each drug class. Pr(>F) is the unadjusted p-value associated with the PERMANOVA F statistic, and FDR is the adjusted p-value to control the false discovery rate.

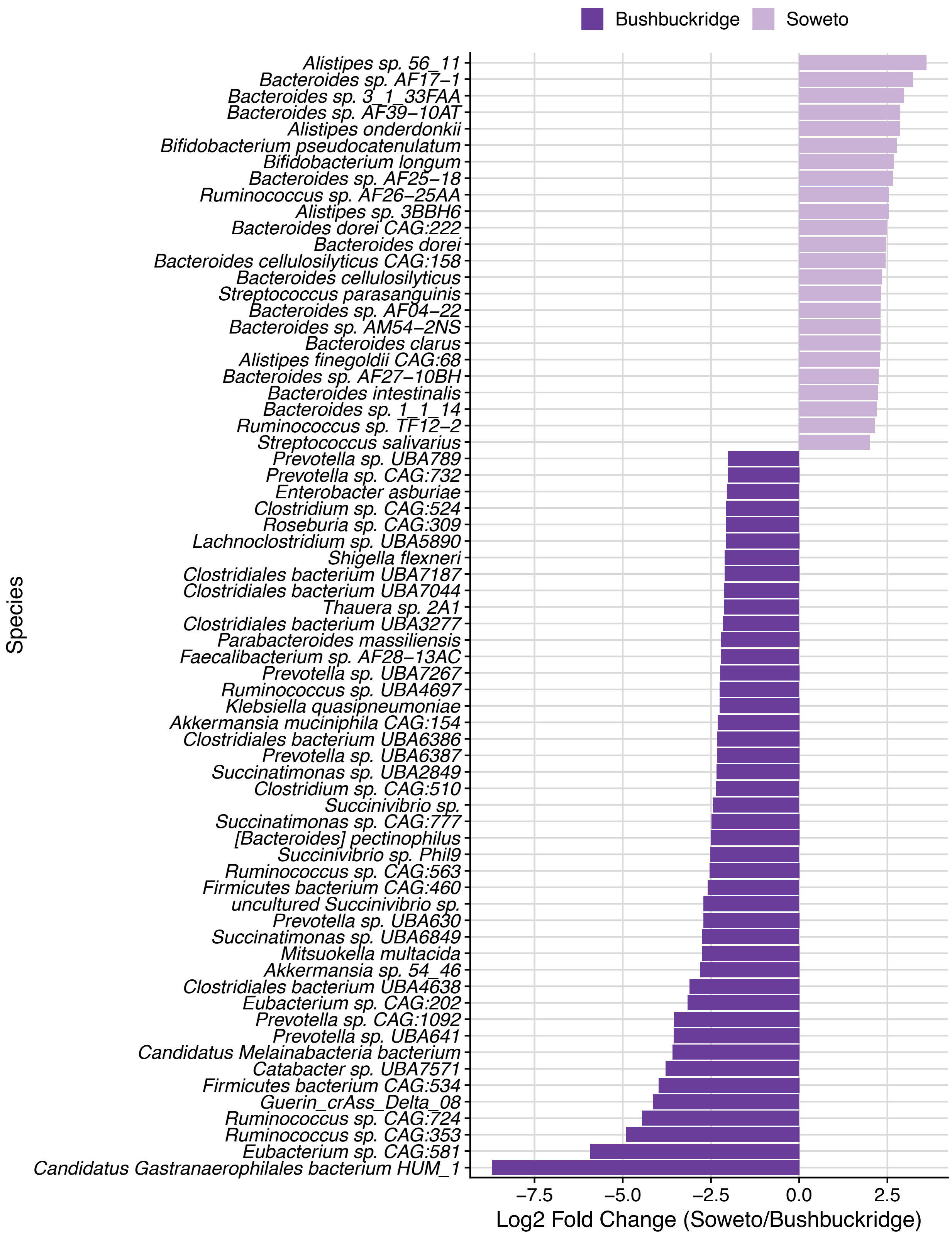
Bushbuckridge
Soweto
Species
Alistipes sp. 56_11
Bacteroides sp. AF17−1
Bacteroides sp. 3_1_33FAA
Bacteroides sp. AF39−10AT
Alistipes onderdonkii
Bifidobacterium pseudocatenulatum
Bifidobacterium longum
Bacteroides sp. AF25−18
Ruminococcus sp. AF26−25AA
Alistipes sp. 3BBH6
Bacteroides dorei CAG:222
Bacteroides dorei
Bacteroides cellulosilyticus CAG:158
Bacteroides cellulosilyticus
Streptococcus parasanguinis
Bacteroides sp. AF04−22
Bacteroides sp. AM54−2NS
Bacteroides clarus
Alistipes finegoldii CAG:68
Bacteroides sp. AF27−10BH
Bacteroides intestinalis
Bacteroides sp. 1_1_14
Ruminococcus sp. TF12−2
Streptococcus salivarius
Prevotella sp. UBA789
Prevotella sp. CAG:732
Enterobacter asburiae
Clostridium sp. CAG:524
Roseburia sp. CAG:309
Lachnoclostridium sp. UBA5890
Shigella flexneri
Clostridiales bacterium UBA7187
Clostridiales bacterium UBA7044
Thauera sp. 2A1
Clostridiales bacterium UBA3277
Parabacteroides massiliensis
Faecalibacterium sp. AF28−13AC
Prevotella sp. UBA7267
Ruminococcus sp. UBA4697
Klebsiella quasipneumoniae
Akkermansia muciniphila CAG:154
Clostridiales bacterium UBA6386
Prevotella sp. UBA6387
Succinatimonas sp. UBA2849
Clostridium sp. CAG:510
Succinivibrio sp.
Succinatimonas sp. CAG:777
[Bacteroides] pectinophilus
Succinivibrio sp. Phil9
Ruminococcus sp. CAG:563
Firmicutes bacterium CAG:460
uncultured Succinivibrio sp.
Prevotella sp. UBA630
Succinatimonas sp. UBA6849
Mitsuokella multacida
Akkermansia sp. 54_46
Clostridiales bacterium UBA4638
Eubacterium sp. CAG:202
Prevotella sp. CAG:1092
Prevotella sp. UBA641
Candidatus Melainabacteria bacterium
Catabacter sp. UBA7571
Firmicutes bacterium CAG:534
Guerin_crAss_Delta_08
Ruminococcus sp. CAG:724
Ruminococcus sp. CAG:353
Eubacterium sp. CAG:581
Candidatus Gastranaerophilales bacterium HUM_1
−7.5
−5.0
−2.5
0.0
2.5
Log2 Fold Change (Soweto/Bushbuckridge)

Supplementary Figure 6. Differentially abundant species between Bushbuckridge and Soweto

Differentially abundant microbial species between rural Bushbuckridge and urban Soweto samples identified by DESeq2. Features with log2 fold change greater than one are shown. Differentially abundant microbial genera are presented in Figure 2c and Supplementary Data 5.

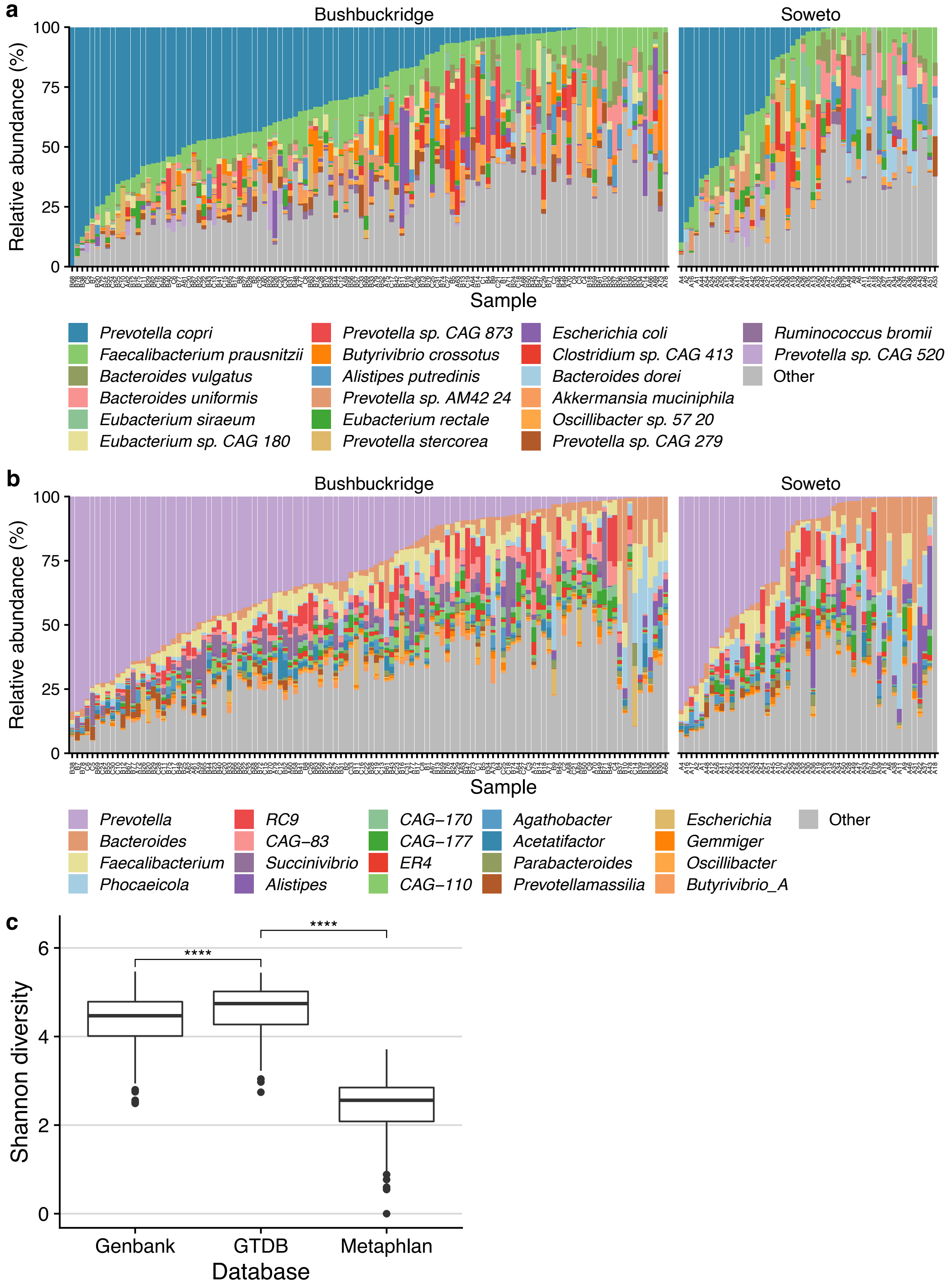
a
Bushbuckridge
Soweto
Relative abundance (%)
100
75
50
25
0
Sample
Prevotella copri
Faecalibacterium prausnitzii
Bacteroides vulgatus
Bacteroides uniformis
Eubacterium siraeum
Eubacterium sp. CAG 180
Prevotella sp. CAG 873
Butyrivibrio crossotus
Alistipes putredinis
Prevotella sp. AM42 24
Eubacterium rectale
Prevotella stercorea
Escherichia coli
Clostridium sp. CAG 413
Bacteroides dorei
Akkermansia muciniphila
Oscillibacter sp. 57 20
Prevotella sp. CAG 279
Ruminococcus bromii
Prevotella sp. CAG 520
Other
b
Bushbuckridge
Soweto
Relative abundance (%)
100
75
50
25
0
Sample
Prevotella
Bacteroides
Faecalibacterium
Phocaeicola
RC9
CAG−83
Succinivibrio
Alistipes
CAG−170
CAG−177
ER4
CAG−110
Agathobacter
Acetatifactor
Parabacteroides
Prevotellamassilia
Escherichia
Gemmiger
Oscillibacter
Butyrivibrio_A
Other
c
Shannon diversity
6
4
2
0
****
****
Genbank
GTDB
Metaphlan
Database

Supplementary Figure 7: GTDB yields increased taxonomic precision and alpha diversity

a Genus-level taxonomy using the MetaPhlAn3 classifier and database.

b Genus-level taxonomy using the Genome Taxonomy Database (GTDB) release 95 and kraken2 classifier.

c Shannon diversity across our custom GenBank database, the GTDB, and MetaPhlAn3 (n=169 samples for each database). Shannon diversity is significantly higher using the GTDB as a reference collection compared to the custom GenBank database (Two-sided Wilcoxon signed rank test, p = 5.738e-05) and MetaPhlAn3 (Two-sided Wilcoxon signed rank test, p < 2.2e-16). Significance values for Wilcoxon signed rank tests denoted in the plot as (****) to represent p < 0.0001. Lower and upper box plot hinges correspond to the first and third quartiles, upper and lower box plot whiskers represent the highest and lowest values within 1.5 times the interquartile range, and the horizontal line represents the median.

a

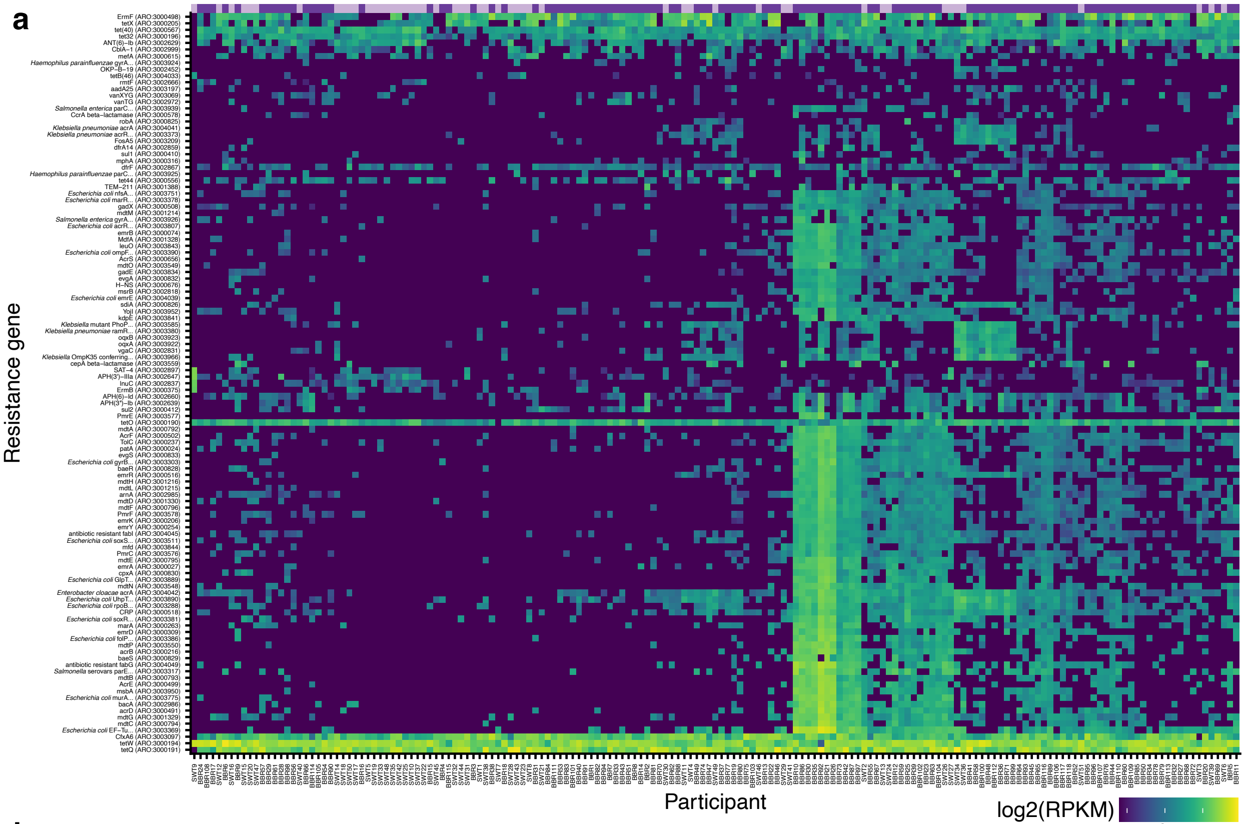

b

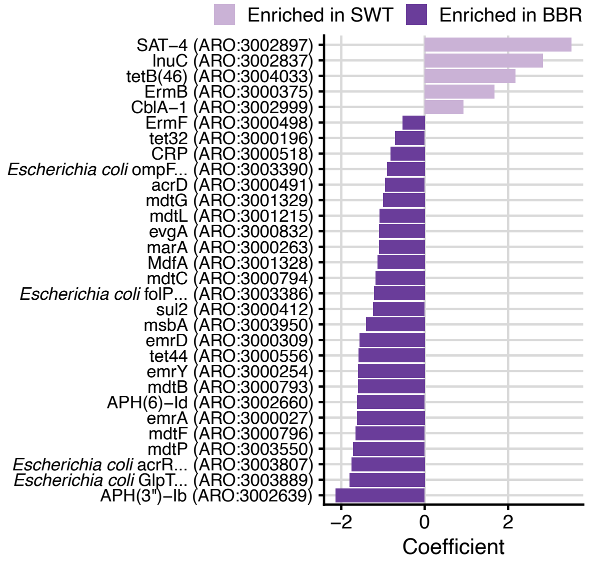

c

Accession	Name
ARO:3003924	*Haemophilus parainfluenzae* gyrA conferring resistance to fluoroquinolones
ARO:3003939	*Salmonella enterica* parC conferring resistance to fluoroquinolones
ARO:3003373	*Klebsiella pneumoniae* acrR with mutation conferring multidrug antibiotic resistance
ARO:3003925	*Haemophilus parainfluenzae* parC conferring resistance to fluoroquinolones
ARO:3003751	*Escherichia coli* nfsA mutations conferring resistance to nitrofurantoin
ARO:3003378	*Escherichia coli* marR mutant conferring antibiotic resistance
ARO:3003926	*Salmonella enterica* gyrA conferring resistance to fluoroquinolones
ARO:3003807	*Escherichia coli* acrR with mutation conferring multidrug antibiotic resistance
ARO:3003390	*Escherichia coli* ompF with mutation
ARO:3003585	*Klebsiella* mutant PhoP conferring antibiotic resistance to colistin
ARO:3003380	*Klebsiella pneumoniae* ramR mutants
ARO:3003966	*Klebsiella* OmpK35 conferring resistance to beta−lactam
ARO:3003303	*Escherichia coli* gyrB conferring resistance to aminocoumarin
ARO:3003511	*Escherichia coli* soxS with mutation conferring antibiotic resistance
ARO:3003889	*Escherichia coli* GlpT with mutation conferring resistance to fosfomycin
ARO:3003890	*Escherichia coli* UhpT with mutation conferring resistance to fosfomycin
ARO:3003288	*Escherichia coli* rpoB mutants conferring resistance to rifampicin
ARO:3003381	*Escherichia coli* soxR with mutation conferring antibiotic resistance
ARO:3003386	*Escherichia coli* folP with mutation conferring resistance to sulfonamides
ARO:3003317	*Salmonella* serovars parE conferring resistance to fluoroquinolones
ARO:3003775	*Escherichia coli* murA with mutation conferring resistance to fosfomycin
ARO:3003369	*Escherichia coli* EF−Tu mutants conferring resistance to Pulvomycin

Supplementary Figure 8: Differentially abundant antibiotic resistance genes between Bushbuckridge and Soweto

Antibiotic resistance genes were profiled using shortBRED against the Comprehensive Antibiotic Resistance Database (CARD). The shortBRED profiles were generated by grouping genes by CARD antibiotic resistance ontology (ARO) accession.

a Heatmap showing log-transformed reads per kilobase per million (RPKM) values for antibiotic resistance genes in the gut metagenome of each participant. Columns (participants) are clustered by Canberra distance, rows (genes) are clustered by Euclidean distance.

b Differentially abundant antibiotic resistance genes in Bushbuckridge (BBR) versus Soweto (SWT). RPKM profiles were compared between study sites using MaAsLin v2 and p-values were adjusted to control the false discovery rate (FDR). Of 113 antibiotic resistance genes tested, 30 with $q < 0.05$ are shown.

c Full CARD names for AROs whose names were truncated for plotting purposes in (**a**) and (**b**).

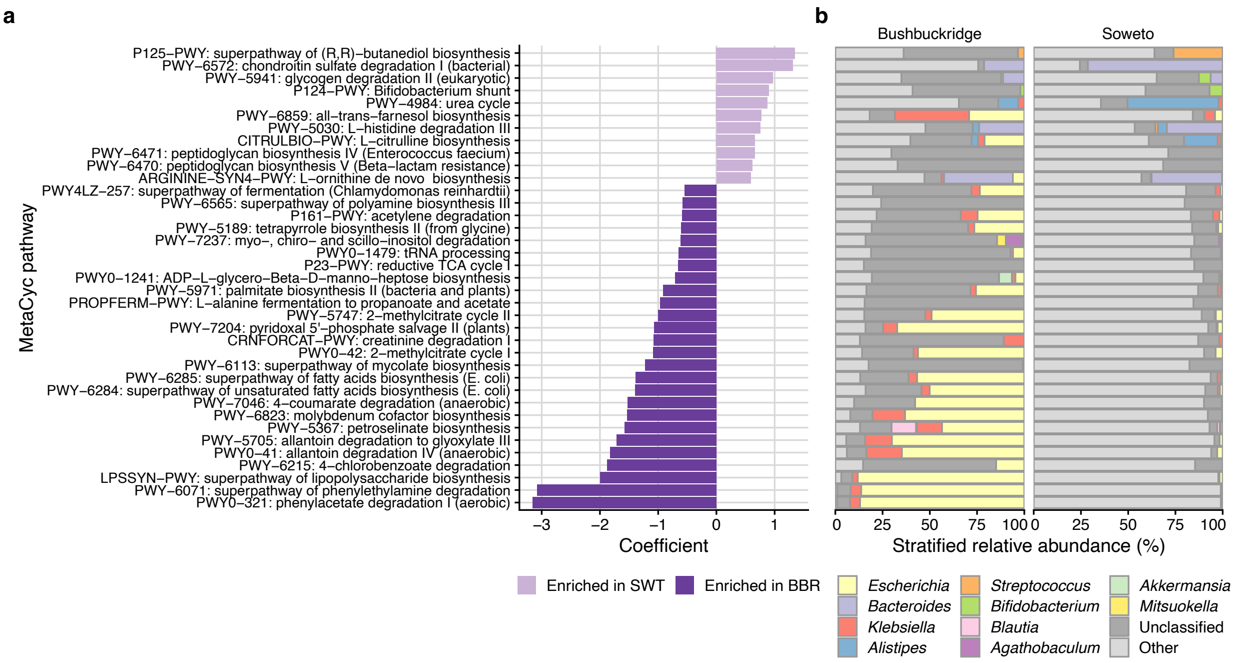

Supplementary Figure 9: Differential MetaCyc pathways between Bushbuckridge and Soweto

a MetaCyc pathways were profiled with HUMAnN v3 and differentially abundant pathways were identified using MaAsLin v2. 424 of 484 pathways (88%) met the 10% prevalence cutoff and 68 of those 424 pathways (16%) were significantly differentially abundant between Bushbuckridge (BBR) and Soweto (SWT) with $q < 0.05$. The 37 pathways with $q < 0.05$ and absolute value of coefficient > 0.5 are shown.

b Stratified pathway composition by taxon for each significant MetaCyc pathway in (**a**).

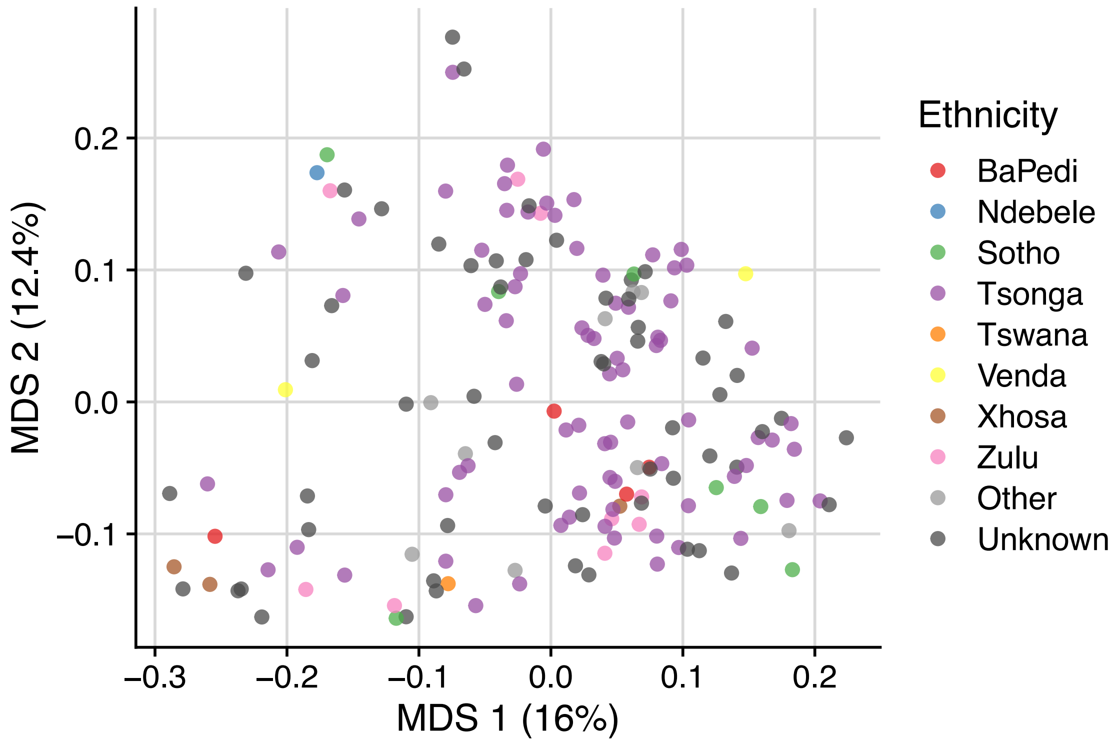

Supplementary Figure 10. South African microbiomes do not cluster by self-reported ethnicity

Multidimensional scaling (MDS) of Bray-Curtis distance with samples colored by self-reported ethnicity. Samples do not cluster by self-reported ethnicity of the participant.

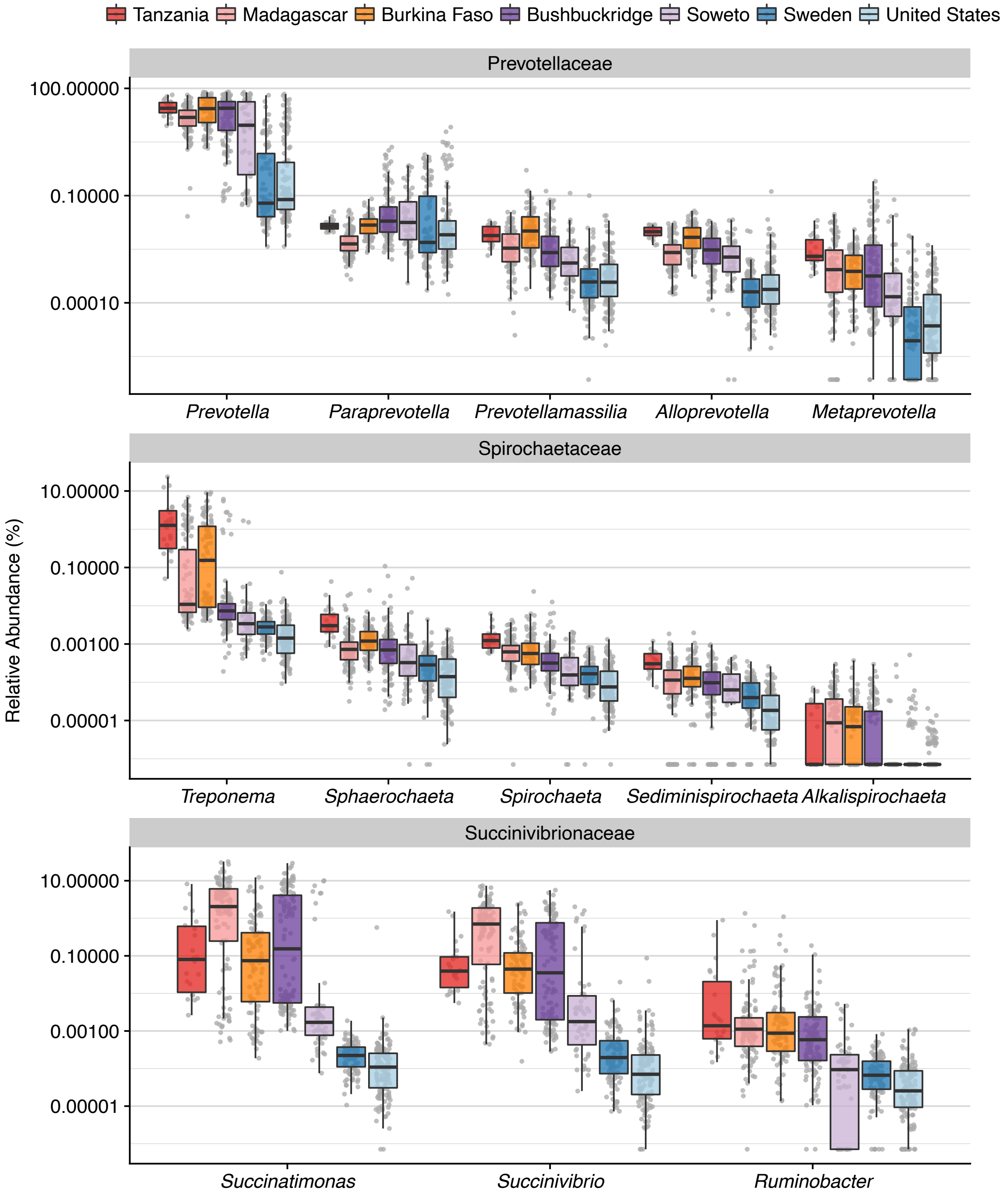

Supplementary Figure 11. Relative abundance of VANISH taxa in global cohort

Relative abundance of VANISH genera from the families Prevotellaceae, Spirochaetaceae, and Succinivibrionaceae. A pseudo-percent was substituted for zero values in order to plot on a log scale. Relative abundance values for most genera trend

toward decreasing from nonwestern cohorts to western cohorts. Box plot lower and upper hinges correspond to the first and third quartiles, upper and lower whiskers represent the highest and lowest values within 1.5 times the interquartile range, and the horizontal line represents the median. Participant sample size: n=22 Tanzania, n=112 Madagascar, n=90 Burkina Faso, n=118 Bushbuckridge, n=51 Soweto, n=100 Sweden, n=134 United States.

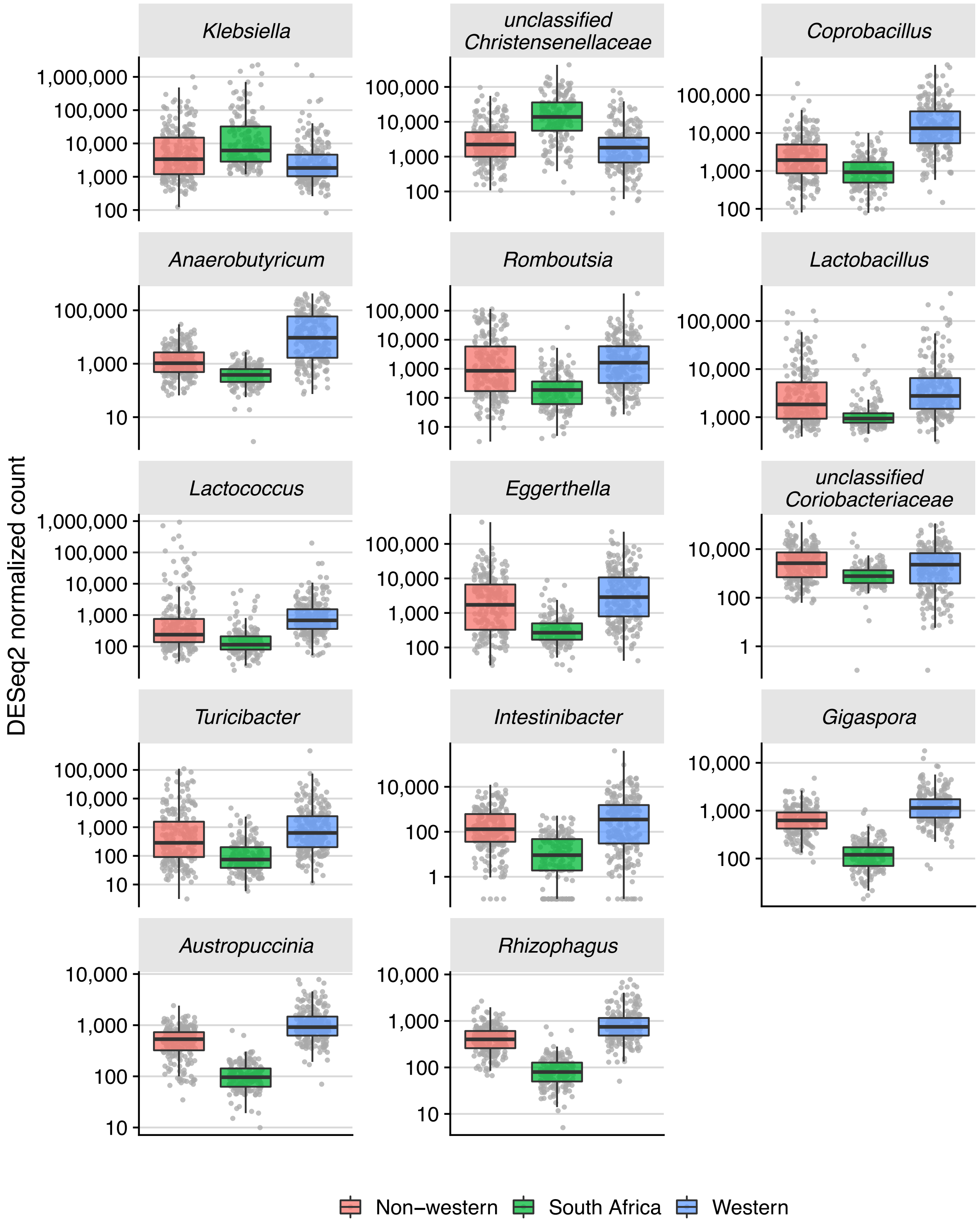

Supplementary Figure 12. Microbial genera enriched or depleted in South Africans relative to other cohorts

Samples were grouped by geographic region into “western” (USA, Sweden; n=234), “nonwestern” (Tanzania, Madagascar, Burkina Faso; n=224) and “South African”

(Bushbuckridge, Soweto; n=169) and genera which distinguish the South African group from the western and nonwestern groups were determined using DESeq2. Genera present with at least 500 counts in 20% of samples were considered (190 features total). 14 features with the same directionality of log2 fold change with respect to South Africa in both comparisons, with a minimum log2 fold change of 2 in each comparison, are shown. A pseudo-percent was added to zero values for plotting. Box plot lower and upper hinges correspond to the first and third quartiles, upper and lower whiskers represent the highest and lowest values within 1.5 times the interquartile range, and the horizontal line represents the median.

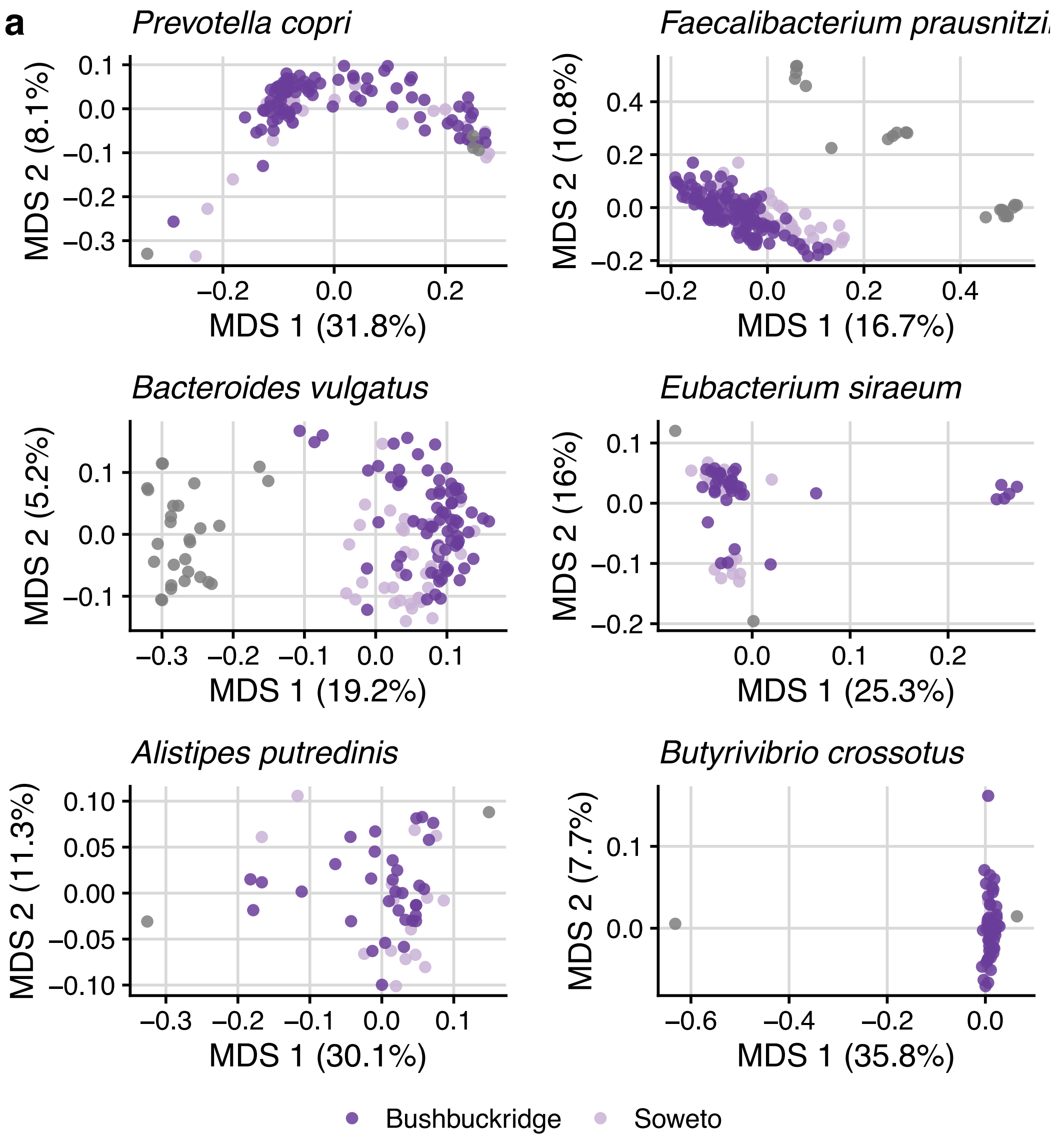

b

Species	R2	Pr(>F)	FDR
Prevotella copri	0.016	0.039	0.0585
Faecalibacterium prausnitzii	0.052	0.001	0.0030
Bacteroides vulgatus	0.027	0.001	0.0030
Eubacterium siraeum	0.046	0.011	0.0220
Alistipes putredinis	0.030	0.099	0.1188
Butyrivibrio crossotus	0.020	0.308	0.3080

Supplementary Figure 13: Pangenomes of South African metagenomic strains

a Multidimensional scaling (MDS) of Jaccard distance between pangenome content of the six most abundant bacteria cohort-wide as measured by MetaPhlAn3.

b PERMANOVA results testing the null hypothesis that the centroids of Bushbuckridge and Soweto sample pangenomes differ in location. PR(>F) signifies the unadjusted p-value for the F statistic and FDR signifies p-values adjusted to control the false discovery rate.

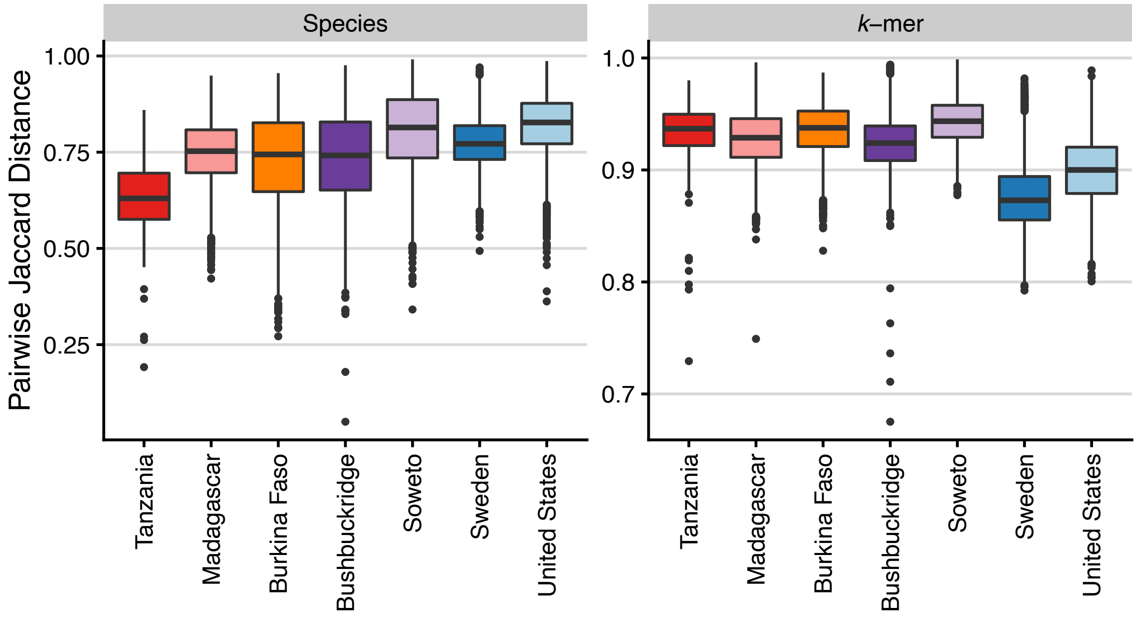

Supplementary Figure 14. Cohort-wise beta diversity computed via Jaccard distance

Comparison of pairwise beta diversity within each cohort based on Jaccard distance of species relative abundance and nucleotide *k*-mer data. Nonwestern populations have greater beta diversity than western populations considering nucleotide *k*-mer composition. Box plot lower and upper hinges correspond to the first and third quartiles, upper and lower whiskers represent the highest and lowest values within 1.5 times the interquartile range, and the horizontal line represents the median. Participant sample size: n=22 Tanzania, n=112 Madagascar, n=90 Burkina Faso, n=118 Bushbuckridge, n=51 Soweto, n=100 Sweden, n=134 United States.

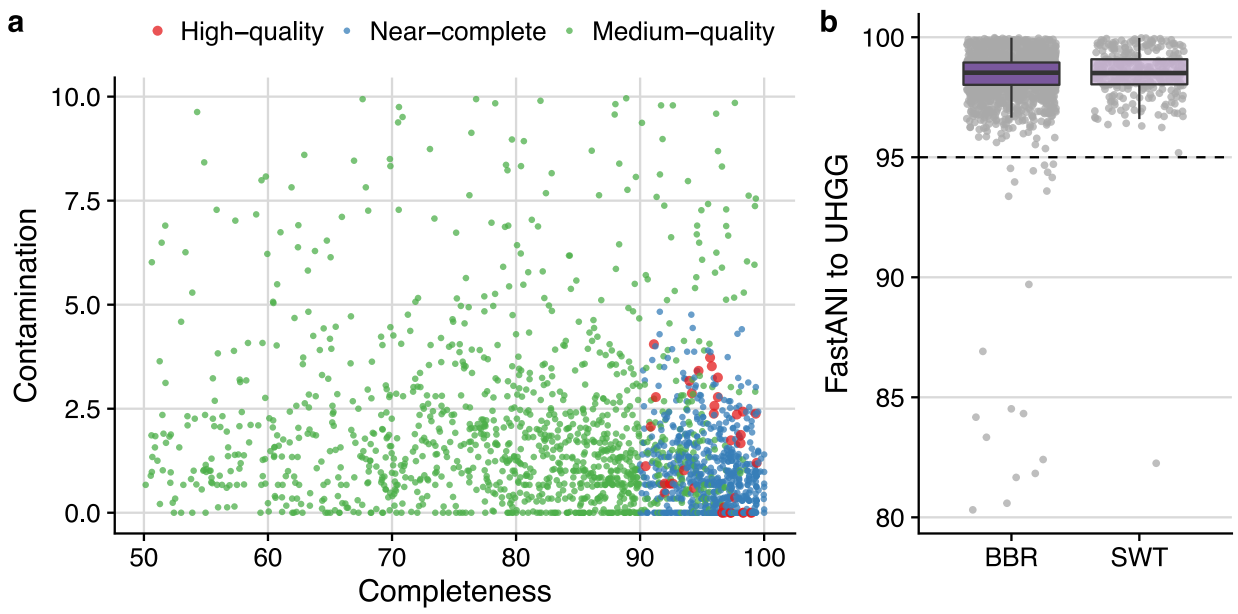

c

Participant	Phylum	Class	Order	Family	Genus	Species
BBR76	Actinobacteriota	Coriobacteriia	Coriobacteriales	Coriobacteriaceae	*Collinsella*	
BBR89	Actinobacteriota	Coriobacteriia	Coriobacteriales	Coriobacteriaceae	*Collinsella*	
BBR96	Actinobacteriota	Coriobacteriia	Coriobacteriales	Coriobacteriaceae	*Collinsella*	
BBR54	Bacteroidota	Bacteroidia	Bacteroidales	P3	*UBA3388*	
BBR98	Desulfobacterota	Desulfovibrionia	Desulfovibrionales	Desulfovibrionaceae	*Mailhella*	*Mailhella massiliensis*
SWT9	Firmicutes	Bacilli	Acholeplasmatales	Anaeroplasmataceae		
BBR38	Firmicutes	Bacilli	RF39	CAG–611	*CAG–877*	
BBR111	Firmicutes_A	Clostridia	Lachnospirales	Lachnospiraceae		
BBR55	Firmicutes_A	Clostridia	Lachnospirales	Lachnospiraceae	*Oribacterium*	
BBR55	Firmicutes_A	Clostridia	Oscillospirales	Oscillospiraceae	*CAG–110*	
BBR106	Firmicutes_A	Clostridia	Peptostreptococcales	Anaerovoracaceae	*UBA1191*	
BBR118	Firmicutes_A	Clostridia	UMGS1810	UMGS1810		
BBR23	Proteobacteria	Alphaproteobacteria	RF32	CAG–239		
BBR106	Verrucomicrobiota	Kiritimatiellae	RFP12	UBA1067	*UBA1067*	
SWT19	Verrucomicrobiota	Verrucomicrobiae	Opitutales	CAG–312	QALA01	

Supplementary Figure 15: Undescribed short-read MAGs

a Distribution of completeness and contamination (as assessed by CheckM) in medium-quality (MQ, n=1,243), near-complete (NC, n=795), and high-quality (HQ, n=39) metagenome-assembled genomes (MAGs) derived from participants in Bushbuckridge (BBR, n=118) and Soweto (SWT, n=51). MQ MAGs must be >50% complete and <10% contaminated; NC MAGs must be ≥90% complete, ≤5% contaminated, and have a contig N50 ≥ 10 kb, average contig length ≥5 kb, ≤500 contigs, and ≥90% of contigs with ≥5X read depth; HQ MAGs must be >90%

complete, <5% contaminated, and have at least 18 tRNA genes and at least one each of the 5S, 16S, and 23S rRNA genes.

b Distribution of FastANI average nucleotide identity values from each MQ or HQ MAG (n=1,773 BBR, n=304 SWT) to the most closely related genome in the Unified Human Gastrointestinal Genome collection (UHGG). Not plotted are ten MQ MAGs with insufficient identity to any genome in UHGG such that a FastANI value could not be calculated. Box plot lower and upper hinges correspond to the first and third quartiles, upper and lower whiskers represent the highest and lowest values within 1.5 times the interquartile range, and the horizontal line represents the median.

c Taxonomic classifications of MAGs from this study with <95% ANI to any genome in UHGG. Classifications according to GTDBtk using release 95 data.

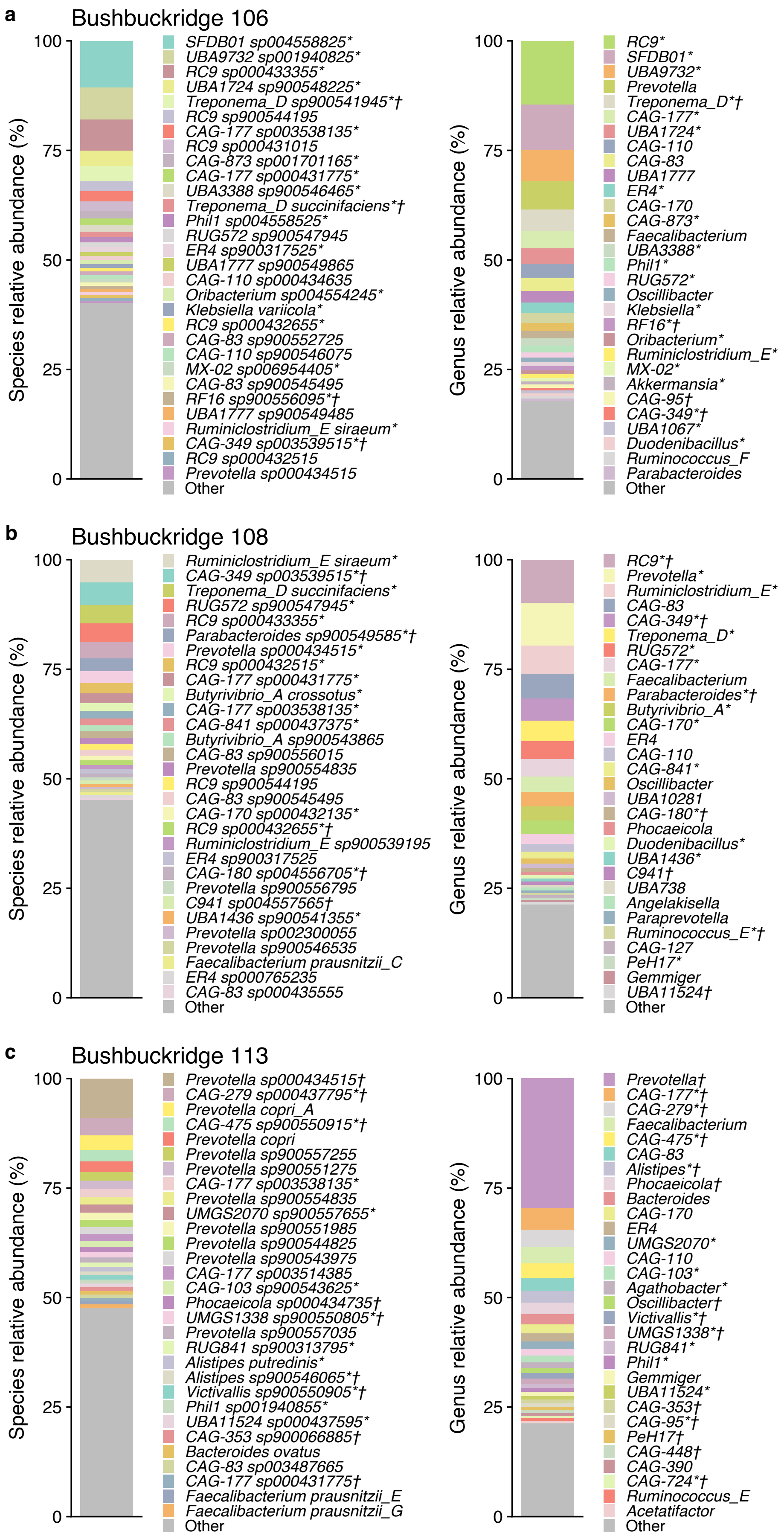

a
Bushbuckridge 106
Species relative abundance (%)
0
25
50
75
100
SFDB01 sp004558825*
UBA9732 sp001940825*
RC9 sp000433355*
UBA1724 sp900548225*
Treponema_D sp900541945*†
RC9 sp900544195
CAG-177 sp003538135*
RC9 sp000431015
CAG-873 sp001701165*
CAG-177 sp000431775*
UBA3388 sp900546465*
Treponema_D succinifaciens*†
Phil1 sp004558525*
RUG572 sp900547945
ER4 sp900317525*
UBA1777 sp900549865
CAG-110 sp000434635
Oribacterium sp004554245*
Klebsiella variicola*
RC9 sp000432655*
CAG-83 sp900552725
CAG-110 sp900546075
MX-02 sp006954405*
CAG-83 sp900545495
RF16 sp900556095*†
UBA1777 sp900549485
Ruminiclostridium_E siraeum*
CAG-349 sp003539515*†
RC9 sp000432515
Prevotella sp000434515
Other
Genus relative abundance (%)
RC9*
SFDB01*
UBA9732*
Prevotella
Treponema_D*†
CAG-177*
UBA1724*
CAG-110
CAG-83
UBA1777
ER4*
CAG-170
CAG-873*
Faecalibacterium
UBA3388*
Phil1*
RUG572*
Oscillibacter
Klebsiella*
RF16*†
Oribacterium*
Ruminiclostridium_E*
MX-02*
Akkermansia*
CAG-95†
CAG-349*†
UBA1067*
Duodenibacillus*
Ruminococcus_F
Parabacteroides
Other
b
Bushbuckridge 108
Species relative abundance (%)
Ruminiclostridium_E siraeum*
CAG-349 sp003539515*†
Treponema_D succinifaciens*
RUG572 sp900547945*
RC9 sp000433355*
Parabacteroides sp900549585*†
Prevotella sp000434515*
RC9 sp000432515*
CAG-177 sp000431775*
Butyrivibrio_A crossotus*
CAG-177 sp003538135*
CAG-841 sp000437375*
Butyrivibrio_A sp900543865
CAG-83 sp900556015
Prevotella sp900554835
RC9 sp900544195
CAG-83 sp900545495
CAG-170 sp000432135*
RC9 sp000432655*†
Ruminiclostridium_E sp900539195
ER4 sp900317525
CAG-180 sp004556705*†
Prevotella sp900556795
C941 sp004557565†
UBA1436 sp900541355*
Prevotella sp002300055
Prevotella sp900546535
Faecalibacterium prausnitzii_C
ER4 sp000765235
CAG-83 sp000435555
Other
Genus relative abundance (%)
RC9*†
Prevotella*
Ruminiclostridium_E*
CAG-83
CAG-349*†
Treponema_D*
RUG572*
CAG-177*
Faecalibacterium
Parabacteroides*†
Butyrivibrio_A*
CAG-170*
ER4
CAG-110
CAG-841*
Oscillibacter
UBA10281
CAG-180*†
Phocaeicola
Duodenibacillus*
UBA1436*
C941†
UBA738
Angelakisella
Paraprevotella
Ruminococcus_E*†
CAG-127
PeH17*
Gemmiger
UBA11524†
Other
c
Bushbuckridge 113
Species relative abundance (%)
Prevotella sp000434515†
CAG-279 sp000437795*†
Prevotella copri_A
CAG-475 sp900550915*†
Prevotella copri
Prevotella sp900557255
Prevotella sp900551275
CAG-177 sp003538135*
Prevotella sp900554835
UMGS2070 sp900557655*
Prevotella sp900551985
Prevotella sp900544825
Prevotella sp900543975
CAG-177 sp003514385
CAG-103 sp900543625*
Phocaeicola sp000434735†
UMGS1338 sp900550805*†
Prevotella sp900557035
RUG841 sp900313795*
Alistipes putredinis*
Alistipes sp900546065*†
Victivallis sp900550905*†
Phil1 sp001940855*
UBA11524 sp000437595*
CAG-353 sp900066885†
Bacteroides ovatus
CAG-83 sp003487665
CAG-177 sp000431775†
Faecalibacterium prausnitzii_E
Faecalibacterium prausnitzii_G
Other
Genus relative abundance (%)
Prevotella†
CAG-177*†
CAG-279*†
Faecalibacterium
CAG-475*†
CAG-83
Alistipes*†
Phocaeicola†
Bacteroides
CAG-170
ER4
UMGS2070*
CAG-110
CAG-103*
Agathobacter*
Oscillibacter†
Victivallis*†
UMGS1338*†
RUG841*
Phil1*
Gemmiger
UBA11524*
CAG-353†
CAG-95*†
PeH17†
CAG-448†
CAG-390
CAG-724*†
Ruminococcus_E
Acetatifactor
Other

Supplementary Figure 16. Taxonomic composition for samples selected for nanopore sequencing

GTDB species-level profiles of short-read data for the three samples selected for Nanopore sequencing: Bushbuckridge 106 (**a**), Bushbuckridge 108 (**b**), and Bushbuckridge 113 (**c**). The top thirty species by mean relative abundance (left panel) and genera (right panel) are shown for each sample. Symbols indicate whether a medium- or high-quality short-read MAG (*) or nanopore MAG (*†*) was recovered for the corresponding taxon.

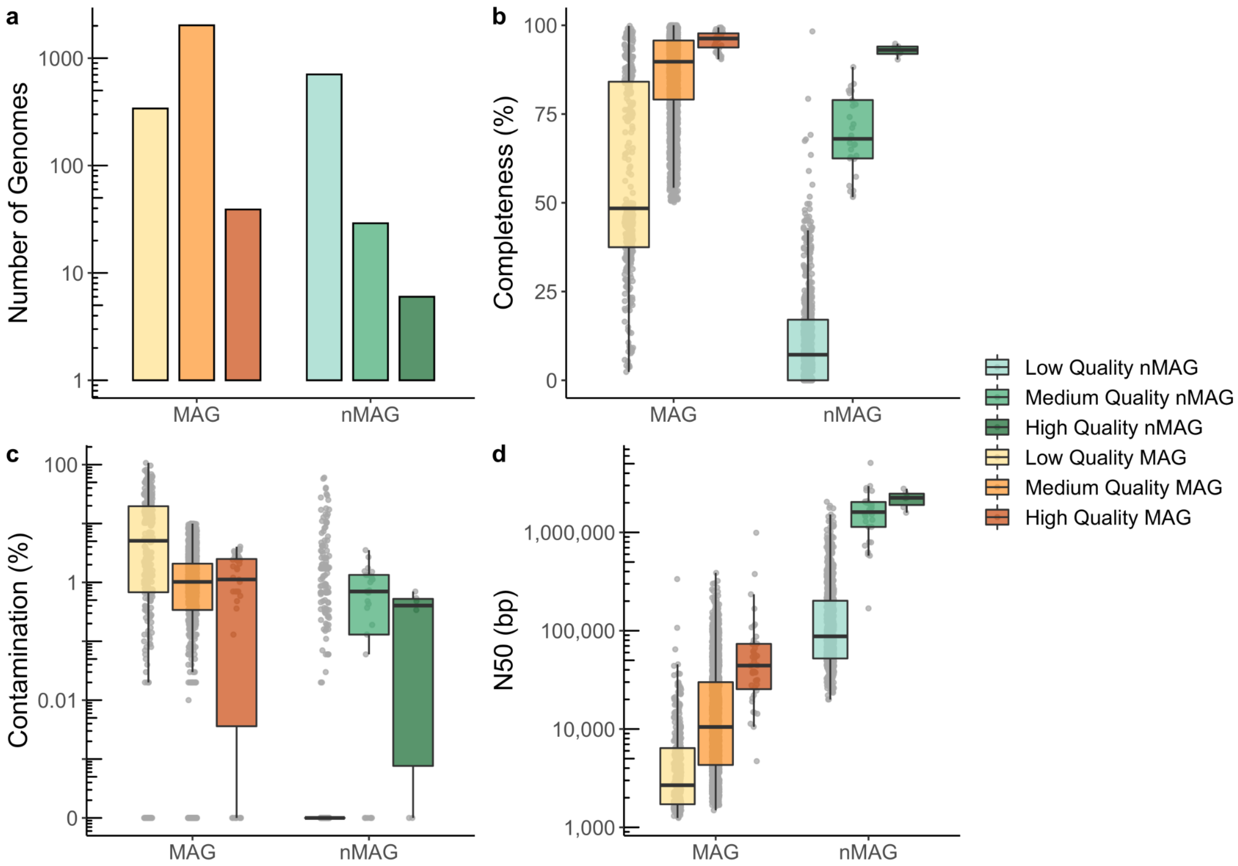

Supplementary Figure 17. Summary statistics for Illumina and nanopore MAGs generated from all samples.

a Number of low-, medium-, and high-quality genomes as evaluated with Bowers et al. standards

b Distribution of MAG percent completeness as determined by CheckM.

c Distribution of MAG percent contamination as determined by CheckM.

d Distribution of MAG N50.

In (**a-d**), box plot lower and upper hinges correspond to the first and third quartiles, upper and lower whiskers represent the highest and lowest values within 1.5 times the interquartile range, and the horizontal line represents the median (n=340 Low Quality MAGs, 2024 Medium Quality MAGs, 39 High Quality MAGs, 706 Low Quality nMAGs, 29 Medium Quality nMAGs, 6 High Quality nMAGs).

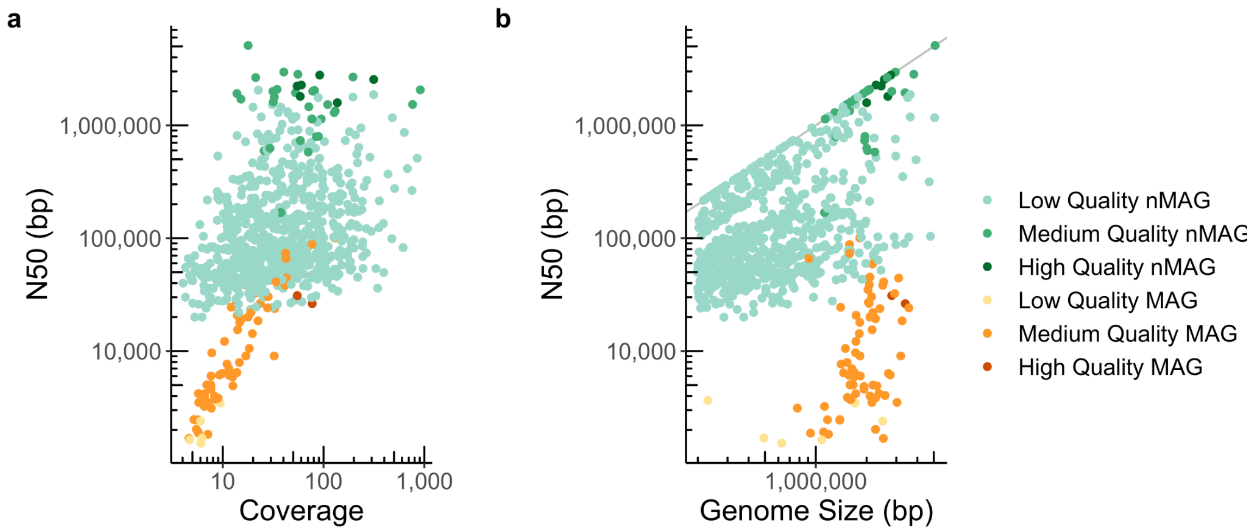

Supplementary Figure 18. Summary statistics of nanopore and short read MAGs generated for three Bushbuckridge samples

a MAG short read or long-read coverage versus MAG N50.

b MAG total size versus MAG N50. Grey line indicates where genome N50 equals total genome size.

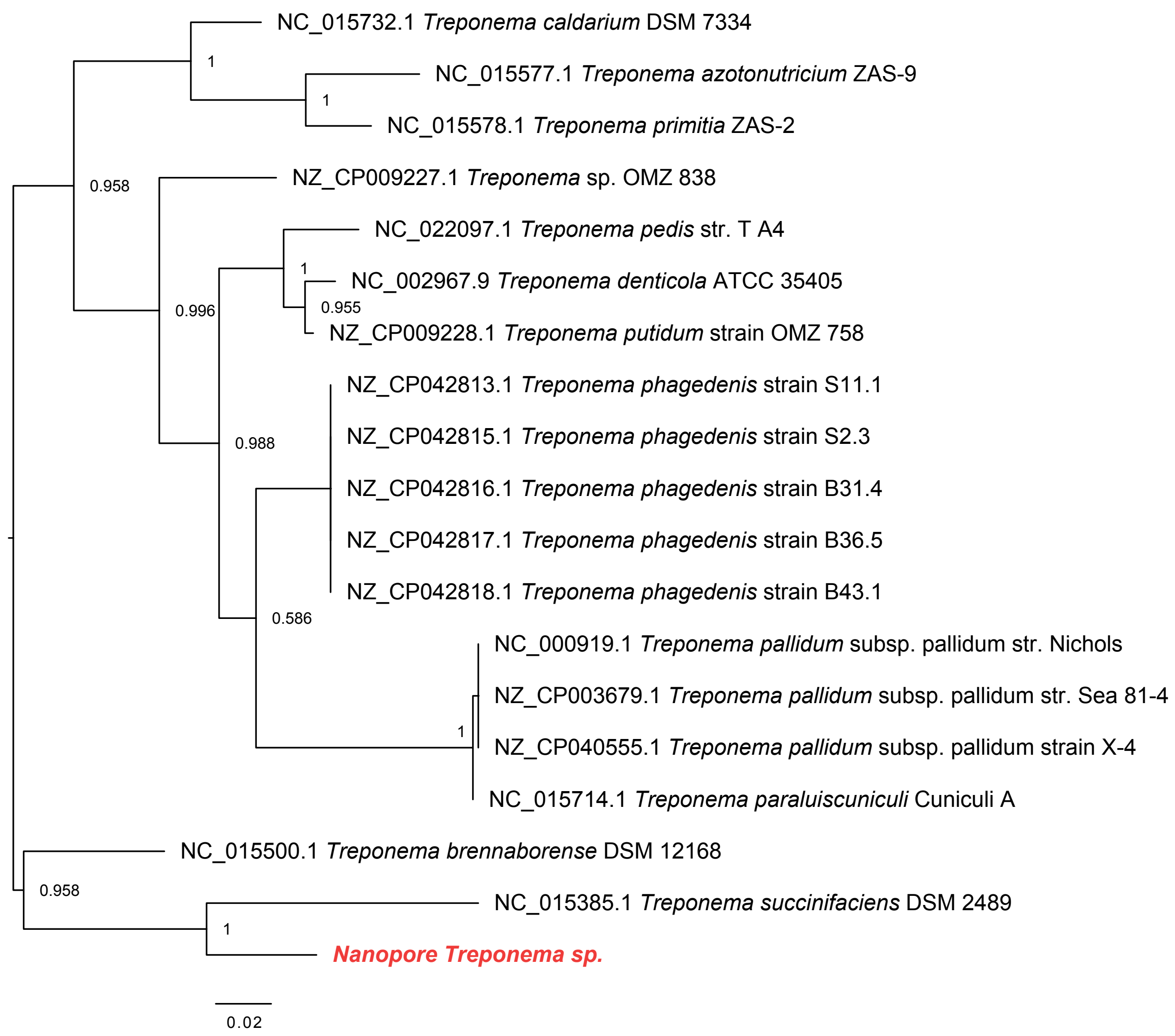

Supplementary Figure 19. Phylogeny of *Treponema* 16S rRNA sequences

Phylogeny of 16S rRNA sequences from species of the genus *Treponema* show that the *Treponema* sp. assembled via Nanopore sequencing is most related to *T. succinifaciens*, but is phylogenetically distinct. Branch labels indicate Shimodaira-Hasegawa support values for splits.

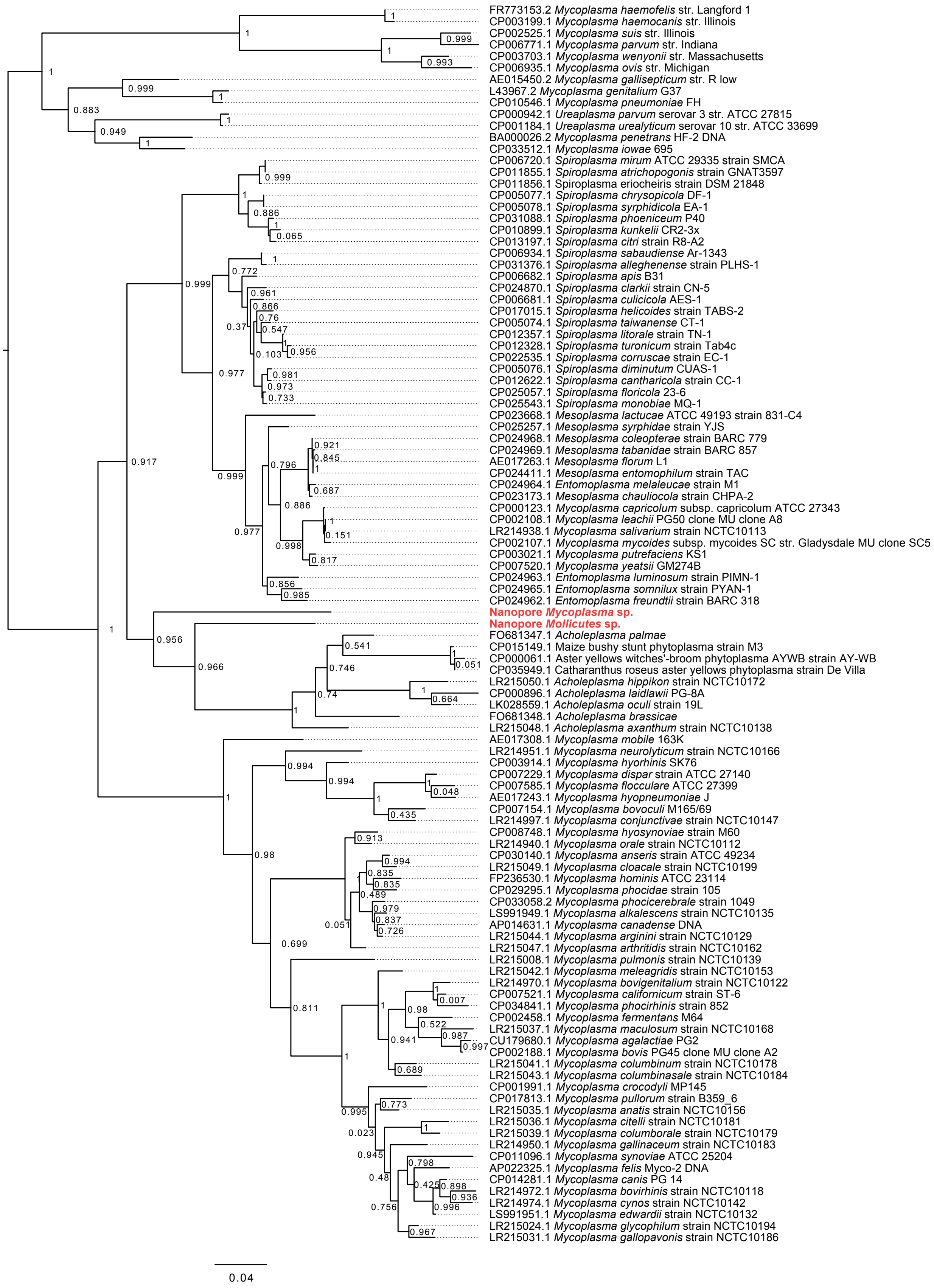
FR773153.2 Mycoplasma haemofelis str. Langford 1
CP003199.1 Mycoplasma haemocanis str. Illinois
CP002525.1 Mycoplasma suis str. Illinois
CP006771.1 Mycoplasma parvum str. Indiana
CP003703.1 Mycoplasma wenyonii str. Massachusetts
CP006935.1 Mycoplasma ovis str. Michigan
AE015450.2 Mycoplasma gallisepticum str. R low
L43967.2 Mycoplasma genitalium G37
CP010546.1 Mycoplasma pneumoniae FH
CP000942.1 Ureaplasma parvum serovar 3 str. ATCC 27815
CP001184.1 Ureaplasma urealyticum serovar 10 str. ATCC 33699
BA000026.2 Mycoplasma penetrans HF-2 DNA
CP033512.1 Mycoplasma iowae 695
CP006720.1 Spiroplasma mirum ATCC 29335 strain SMCA
CP011855.1 Spiroplasma atrichopogonis strain GNAT3597
CP011856.1 Spiroplasma eriocheiris strain DSM 21848
CP005077.1 Spiroplasma chrysopicola DF-1
CP005078.1 Spiroplasma syrphidicola EA-1
CP031088.1 Spiroplasma phoeniceum P40
CP010899.1 Spiroplasma kunkelii CR2-3x
CP013197.1 Spiroplasma citri strain R8-A2
CP006934.1 Spiroplasma sabaudiense Ar-1343
CP031376.1 Spiroplasma alleghenense strain PLHS-1
CP006682.1 Spiroplasma apis B31
CP024870.1 Spiroplasma clarkii strain CN-5
CP006681.1 Spiroplasma culicicola AES-1
CP017015.1 Spiroplasma helicoides strain TABS-2
CP005074.1 Spiroplasma taiwanense CT-1
CP012357.1 Spiroplasma litorale strain TN-1
CP012328.1 Spiroplasma turonicum strain Tab4c
CP022535.1 Spiroplasma corruscae strain EC-1
CP005076.1 Spiroplasma diminutum CUAS-1
CP012622.1 Spiroplasma cantharicola strain CC-1
CP025057.1 Spiroplasma floricola 23-6
CP025543.1 Spiroplasma monobiae MQ-1
CP023668.1 Mesoplasma lactucae ATCC 49193 strain 831-C4
CP025257.1 Mesoplasma syrphidae strain YJS
CP024968.1 Mesoplasma coleopterae strain BARC 779
CP024969.1 Mesoplasma tabanidae strain BARC 857
AE017263.1 Mesoplasma florum L1
CP024411.1 Mesoplasma entomophilum strain TAC
CP024964.1 Entomoplasma melaleucae strain M1
CP023173.1 Mesoplasma chauliocola strain CHPA-2
CP000123.1 Mycoplasma capricolum subsp. capricolum ATCC 27343
CP002108.1 Mycoplasma leachii PG50 clone MU clone A8
LR214938.1 Mycoplasma salivarium strain NCTC10113
CP002107.1 Mycoplasma mycoides subsp. mycoides SC str. Gladysdale MU clone SC5
CP003021.1 Mycoplasma putrefaciens KS1
CP007520.1 Mycoplasma yeatsii GM274B
CP024963.1 Entomoplasma luminosum strain PIMN-1
CP024965.1 Entomoplasma somnilux strain PYAN-1
CP024962.1 Entomoplasma freundtii strain BARC 318
Nanopore Mycoplasma sp.
Nanopore Mollicutes sp.
FO681347.1 Acholeplasma palmae
CP015149.1 Maize bushy stunt phytoplasma strain M3
CP000061.1 Aster yellows witches'-broom phytoplasma AYWB strain AY-WB
CP035949.1 Catharanthus roseus aster yellows phytoplasma strain De Villa
LR215050.1 Acholeplasma hippikon strain NCTC10172
CP000896.1 Acholeplasma laidlawii PG-8A
LK028559.1 Acholeplasma oculi strain 19L
FO681348.1 Acholeplasma brassicae
LR215048.1 Acholeplasma axanthum strain NCTC10138
AE017308.1 Mycoplasma mobile 163K
LR214951.1 Mycoplasma neurolyticum strain NCTC10166
CP003914.1 Mycoplasma hyorhinis SK76
CP007229.1 Mycoplasma dispar strain ATCC 27140
CP007585.1 Mycoplasma flocculare ATCC 27399
AE017243.1 Mycoplasma hyopneumoniae J
CP007154.1 Mycoplasma bovoculi M165/69
LR214997.1 Mycoplasma conjunctivae strain NCTC10147
CP008748.1 Mycoplasma hyosynoviae strain M60
LR214940.1 Mycoplasma orale strain NCTC10112
CP030140.1 Mycoplasma anseris strain ATCC 49234
LR215049.1 Mycoplasma cloacale strain NCTC10199
FP236530.1 Mycoplasma hominis ATCC 23114
CP029295.1 Mycoplasma phocidae strain 105
CP033058.2 Mycoplasma phocicerebrale strain 1049
LS991949.1 Mycoplasma alkalescens strain NCTC10135
AP014631.1 Mycoplasma canadense DNA
LR215044.1 Mycoplasma arginini strain NCTC10129
LR215047.1 Mycoplasma arthritidis strain NCTC10162
LR215008.1 Mycoplasma pulmonis strain NCTC10139
LR215042.1 Mycoplasma meleagridis strain NCTC10153
LR214970.1 Mycoplasma bovigenitalium strain NCTC10122
CP007521.1 Mycoplasma californicum strain ST-6
CP034841.1 Mycoplasma phocirhinis strain 852
CP002458.1 Mycoplasma fermentans M64
LR215037.1 Mycoplasma maculosum strain NCTC10168
CU179680.1 Mycoplasma agalactiae PG2
CP002188.1 Mycoplasma bovis PG45 clone MU clone A2
LR215041.1 Mycoplasma columbinum strain NCTC10178
LR215043.1 Mycoplasma columbinasale strain NCTC10184
CP001991.1 Mycoplasma crocodyli MP145
CP017813.1 Mycoplasma pullorum strain B359_6
LR215035.1 Mycoplasma anatis strain NCTC10156
LR215036.1 Mycoplasma citelli strain NCTC10181
LR215039.1 Mycoplasma columborale strain NCTC10179
LR214950.1 Mycoplasma gallinaceum strain NCTC10183
CP011096.1 Mycoplasma synoviae ATCC 25204
AP022325.1 Mycoplasma felis Myco-2 DNA
CP014281.1 Mycoplasma canis PG 14
LR214972.1 Mycoplasma bovirhinis strain NCTC10118
LR214974.1 Mycoplasma cynos strain NCTC10142
LS991951.1 Mycoplasma edwardii strain NCTC10132
LR215024.1 Mycoplasma glycophilum strain NCTC10194
LR215031.1 Mycoplasma gallopavonis strain NCTC10186
0.04

Supplementary Figure 20. Phylogeny of Mollicutes 16S rRNA sequences

Phylogeny of 16S rRNA sequences from species of the class Mollicutes showing the Mollicutes and Mycoplasma genomes assembled via nanopore sequencing. Branch labels indicate Shimodaira-Hasegawa support values for splits.

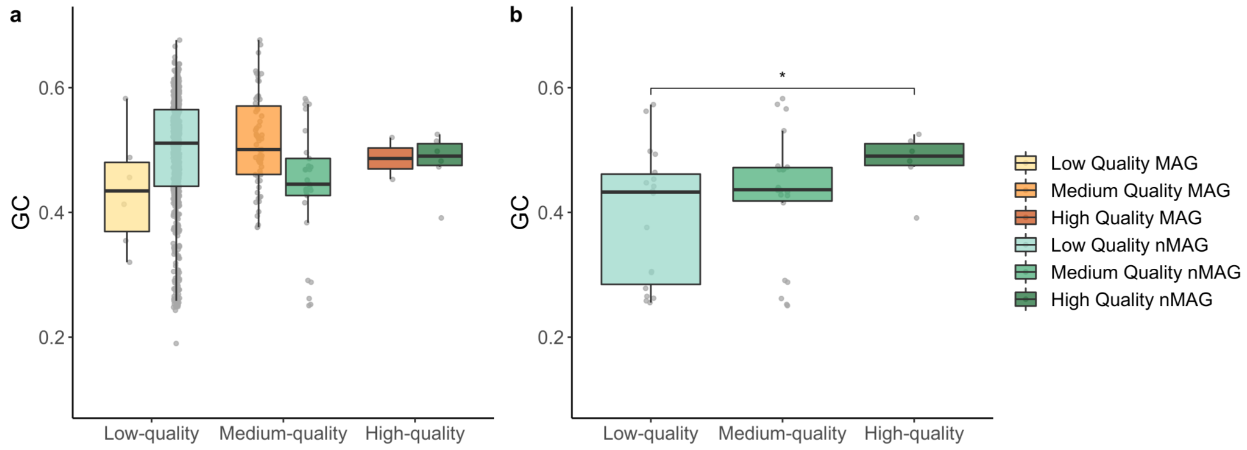

Supplementary Figure 21. GC content of MAGs and nMAGs generated from three Bushbuckridge samples

a GC content range of MAGs and nMAGs. n=6 Low Quality MAGs, 67 Medium Quality MAGs, 2 High Quality MAGs, 706 Low Quality nMAGs, 29 Medium Quality nMAGs, 6 High Quality nMAGs.

b nMAGs with contig N50 values greater than one megabase. GC content of low-quality nMAGs is lower than the GC content of high-quality nMAGs, despite nMAGs of all quality having N50 values of higher than one megabase. (*) denotes p = 0.047, two-sided Wilcoxon rank sum test. n=18 Low Quality nMAGs, 22 Medium Quality nMAGs, 6 High Quality nMAGs.

In (**a**) and (**b**), box plot lower and upper hinges correspond to the first and third quartiles, upper and lower whiskers represent the highest and lowest values within 1.5 times the interquartile range, and the horizontal line represents the median.

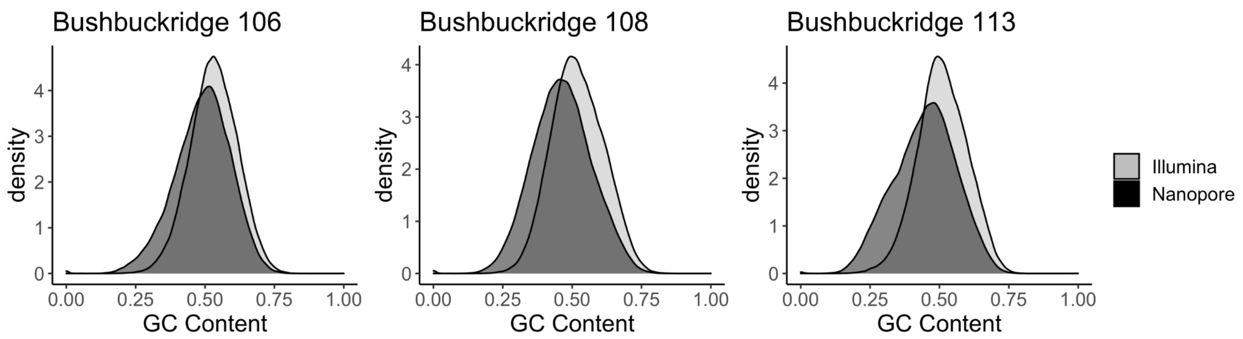

Supplementary Figure 22. GC content of nanopore and Illumina sequencing reads generated from three Bushbuckridge samples

GC content was calculated for all processed Illumina reads (average length of 126 bp) and for 126 bp windows of all nanopore reads. GC content distribution was subsampled to 100,000 measurements per method.

Supplementary Tables

Supplementary Table 1. Concomitant medications

**Medicine category**	**Medicine name**	**Number of participants**
ANALGESIC	CHLORZOXAZONE/DICLOFENAC/PARACETAMOL	1
	PARACETAMOL	8
ANTACID	MAGNESIUM-CARBONATE/MAGNESIUM-TRISILLICATE/SODIUM-BICARBONATE	1
	RANITIDINE	1
ANTI-DIARRHEAL	LOPERAMIDE	2
ANTI-EPILEPTIC	CARBAMAZEPINE	1
ANTI-HISTAMINE	CHLORPHENIRAMINE	1
ANTI-HYPERGLYCEMIC	GLIBENCLAMIDE	2
	METFORMIN	6
ANTI-HYPERTENSIVE	AMILORIDE-HYDROCHLORIDE/HYDROCHLOROTHIAZIDE	2
	AMLODIPINE	10
	CARVEDILOL	1
	DIGOXIN	2
	ENALAPRIL	13
	HYDROCHLOROTHIAZIDE	25
ANTI-PARKINSONIAN	PHENERINE	2
ANTI-PSYCHOTIC	HALOPERIDOL	2
ANTI-SPASMODIC	HYOSCINE-BUTYLBROMIDE	1
ANTIBIOTIC	AMOXICILLIN	5
	CEFIXIME	1
	CO-TRIMOXAZOLE	1
	METRONIDAZOLE	3
CORTICOSTEROID	PREDNISONE	1
DIURETIC	FUROSEMIDE	2
HORMONE	LEVOTHYROXINE	1
INSULIN	INSULIN	1
NSAID	ASPIRIN	13
	CELECOXIB	1
	DICLOFENAC	2
	INDOMETACIN	2
PROTON-PUMP-INHIBITOR	LANSOPRAZOLE	2
STATIN	SIMVASTATIN	2
SUPPLEMENT	CALCIUM	1
	PYRIDOXINE	2
OTHER	GLUCOSAMINE-SULFATE	1
	POTASSIUM-CHLORIDE	1

Supplementary Table 2. Study site characteristics as of 2011 census

	**Bushbuckridge**	**Soweto**
**Total population**	541,248	1,271,628
**Population density**	53 persons per km^2	6,357 persons per km^2
**Number of households**	134,197	355,331
**Unemployment rate**	52.10%	*Not available*
**Higher education aged 20+**	7.40%	9.30%
**Households with flush toilet connected to sewerage**	6.80%	91.60%
**Households with piped water inside dwelling**	11.90%	55%
**Households with electricity for lighting**	93.90%	93.10%

Supplementary Table 3. Species associated with human genetic data

Genus	SNP	Gene/Region	β	p
Alistipes sp. CAG 435	rs35894506	FOXP1	-3.2	2.60E-08
	rs11197532	GFRA1	2.9	3.70E-08
Bacteroides fragilis	rs67898769	FOXP1	1.9	2.60E-08
	rs10137347	reg: RNASE6	1.23	4.90E-08
Faecalibacterium prausnitzii	rs7641190	lincRNA	-0.5	4.40E-08
	rs7641364	lincRNA	-0.5	4.40E-08
Prevotella-sp-AM23-5	rs3091514	SLC2A10	-2	1.70E-08
Prevotella-sp-TF12-30	rs3091514	SLC2A10	-2	3.90E-08
Ruminococcaceae bacterium	rs145200779	lincRNA	-1.5	2.20E-08
	rs77253663	intergenic	1.2	1.60E-08
	rs13360502	LINC01484/intron	-1.44	3.50E-08
	rs116133146	LINC01484/downstream transcript	-1.44	3.50E-08
	rs78133191	NTRK2	-1.5481	4.12E-08
	rs7873546	NTRK2	-1.5481	4.12E-08

Significance evaluated using GEMMA, using 5x10^-8 as the p-value to account for multiple testing both with multiple SNPs and microbiome genera

Supplementary Table 4. Cohorts analyzed in global microbiome comparison

Reference	Study	Geography	Lifestyle	Sequencing strategy	Participants	Public database	Accession
HMP Consortium. Nature (2012)	Structure, function and diversity of the healthy human microbiome	USA	Urban	101 bp paired-end reads, Illumina GAIIx	134	NCBI SRA	PRJNA43017
Rampelli *et al.* Current Biology (2015)	Metagenome Sequencing of the Hadza Hunter-Gatherer Gut Microbiota	Tanzania	Hunter-gatherer	101 bp paired-end reads, Illumina GAIIx	22	NCBI SRA	PRJNA278393
Pasolli *et al.* Cell Host and Microbe (2019)	Distinct Genetic and Functional Traits of Human Intestinal Prevotella copri Strains Are Associated with Different Habitual Diets	Madagascar	Agriculturalist/ hunter-gatherer	101 bp paired-end reads, Illumina HiSeq	112	NCBI SRA	PRJNA485056
Backhed *et al.* Cell Host and Microbe (2015)	Dynamics and stabilization of the human gut microbiome during the first year of life	Sweden	Urban	101 bp paired-end reads, Illumina HiSeq 2000	100	European Nucleotide Archive	ERP005989
Jacobson *et al.* Nature Scientific Reports (2021)	Analysis of global human gut metagenomes shows that metabolic resilience potential for short-chain fatty acid production is strongly influenced by lifestyle	Burkina Faso	Agriculturalist	150 bp paired-end reads, Illumina NovaSeq S1	90	NCBI SRA	PRJNA690543
This study	This study	South Africa	Rural and urban cohorts	150 bp paired-end reads, Illumina HiSeq 4000	169	NCBI SRA	PRJNA678454

Supplementary Table 5. Nanopore sequencing statistics of three Bushbuckridge samples

	BBR106	BBR108	BBR113
**Number of reads**	4576442	7067362	3776000
**Read length N50 (bp)**	9429	5541	9855
**Total Gbp**	22.78	20.8	15.54
**nMAGs**	208	258	275
**Average nMAG N50 (bp)**	220619.8	209291	341311.9
**Filtered, non-redundant nMAGs**	6	8	21
**Medium-quality nMAGs**	4	7	18
**High-quality nMAGs**	2	1	3

Community engagement

It is an ethical imperative to include diverse and understudied populations in biomedical research, and it is of utmost importance to conduct research equitably in groups which may be socioeconomically vulnerable. During the course of this project, researchers traveled to field sites in both Soweto and Bushbuckridge on multiple occasions, including a trip to the Agincourt Health and Demographic Surveillance Site (HDSS) early in the planning phases of the study where researchers met with members of the Community Advisory Group (CAG). This team of community members from across the Bushbuckridge Municipality routinely meets with researchers to discuss and approve research projects in the community and voice questions and concerns on behalf of the community. The research team sought the opinion of the CAG as to whether the study of stool would be appropriate in the community, given that attitudes toward collection of fecal samples may differ across cultures. The CAG expressed that it would be appropriate to collect stool if it was explained clearly to participants that the stool was for scientific purposes, that it would not be used to cause harm in any way to participants, and that it would not be sold.

On a second visit to the Agincourt HDSS, the research team conducted a microbiome workshop for community members to attend to learn more about how studying the gut microbiome may improve understanding of the health of African populations. Community members learned about bacteria, their role in human health, and the ways in which studying gut microbiome composition might impact our understanding of human health in the region. Community members were given the opportunity to ask questions and discuss the research project.

Additional information on study sites

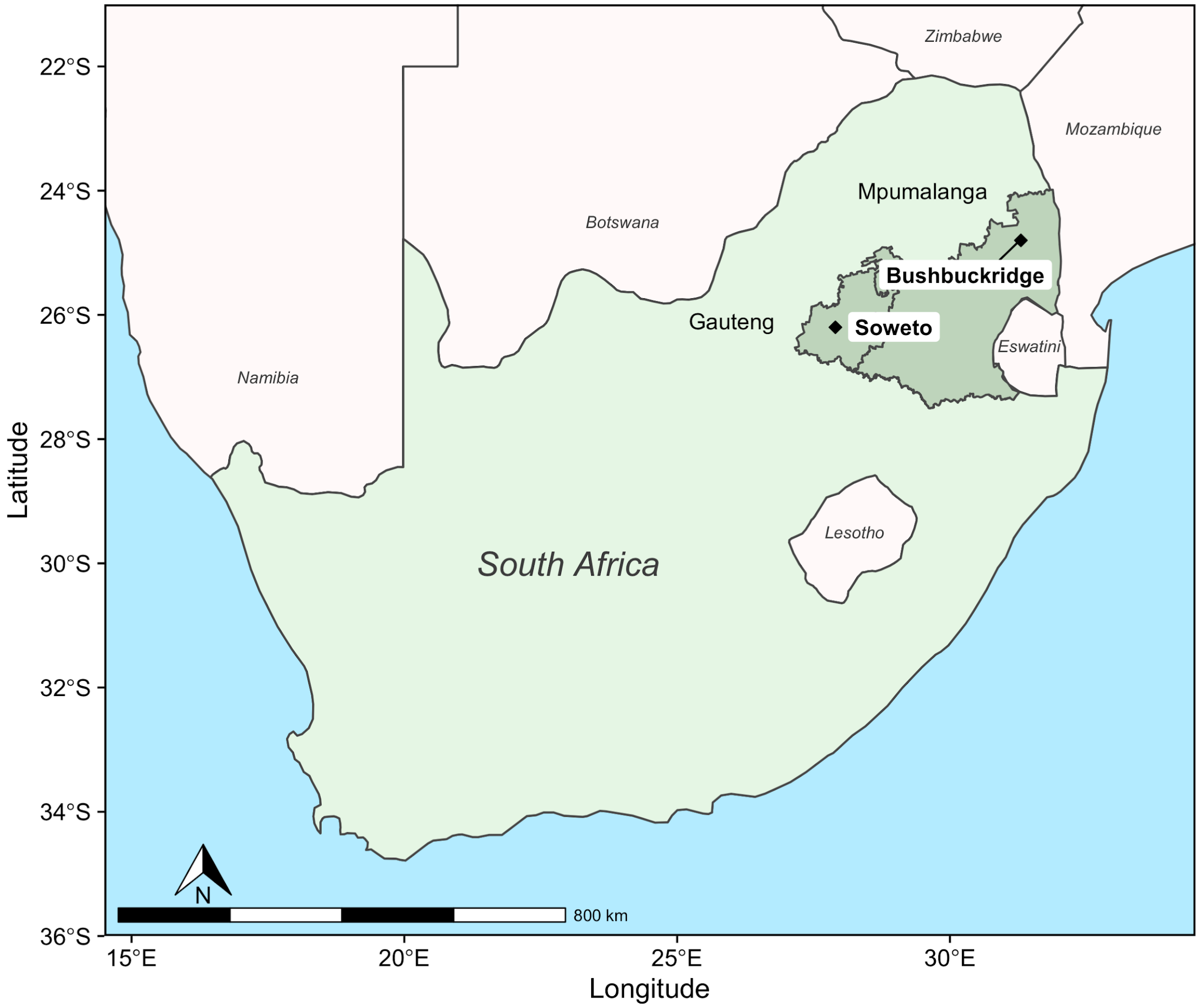

The University of the Witwatersrand in Johannesburg, South Africa has long-standing partnerships with health and demographic surveillance sites (HDSSs) across South Africa. The Agincourt HDSS, run by the MRC/Wits Rural Public Health and Health Transitions Research Unit surveys individuals living in the Bushbuckridge Municipality in rural Mpumalanga province. Bushbuckridge Municipality is the largest municipality in Mpumalanga by population and by land area (10,250 km^2), and includes areas of Kruger National Park. The MRC/Wits Developmental Pathways for Health Research Unit surveys individuals living in urban Soweto, a township of 200.03 km^2 that is part of Johannesburg in Gauteng province. Soweto's name is derived from **So**uth **We**stern **To**wnships and is formally incorporated in the city of Johannesburg.

The populations of both Bushbuckridge and Soweto are predominantly black African (http://www.statssa.gov.za)

As a result of HDSS surveillance, much is known about changes in disease risk and causes of death over time in Bushbuckridge and Soweto. For instance, rates of obesity are higher in women compared to men in both Bushbuckridge and Soweto HDSS areas[1,2].

Additionally, diet and lifestyle in Bushbuckridge and Soweto have been extensively surveyed. Pisa *et al.*[3] administered food frequency questionnaires to adolescents living within the Agincourt HDSS (Bushbuckridge) and identified nutrient patterns via principal components analysis that explain 79% of the variance in nutritional intake in the study population. They found that female gender and being in the lowest socioeconomic status (SES) tertile associated with animal driven nutrients, being in mid-puberty associated with vitamins, fiber, and vegetable oil nutrients, and physical activity and being in the lowest SES tertile associated with mixed diet driven nutrients. Additionally, body mass index (BMI) was found to associate with nutritional patterns. Sedibe *et al.*[4] surveyed dietary practices and rates of obesity in adolescents from Bushbuckridge and Soweto and found that across study sites, participants regularly consumed fast foods. Rural and/or male participants were less likely to be overweight and obese than females, and irregular consumption of breakfast on weekdays was associated with increased risk of overweight and obesity. Additionally, a qualitative study of adolescent females in Bushbuckridge described dietary practices[5]. Findings included the observation that pap (maize porridge) and tea are common breakfast options among the population of Bushbuckridge, and that leafy vegetables, legumes, and nuts are viewed as healthy by participants but affordability can be a barrier to access, and fruit is not easily accessible. Participants described how limitations in household resources restricted their options for purchasing healthy foods at school, and that instead they bought cheaper options including bread, vetkoek (fried bread), kota (white bread filled with potato chips and processed meat or cheese) or other processed snack foods. In adult South Africans participating in AWI-Gen, consumption of sugar-sweetened beverages was positively associated with BMI in women[6].

Microbiome and human genetic association testing

To test whether Bray-Curtis distance was correlated with genetic distance, we used 114 samples from Bushbuckridge for whom QC had passed on both human and microbiome data. This is a low *n*, so the absence of correlation in this study is only weak evidence of a general claim of lack of correlation. We did not include the Soweto data because the different environment would have been a confounding factor. All participants in the AWI-Gen study have been genotyped using the H3A Custom Array, an approximately 2.2 million SNP array designed to maximise coverage in African populations (https://h3abionet.org/h3africa-chip). QC of the genotype data was done using the H3ABioNet H3AGWAS QC pipeline (https://github.com/h3abionet/h3agwas). Genetic relatedness (PI-HAT) was computed using PLINK[7] and converted to a distance score. We also computed principal components using PLINK. Data for our participants was extracted. There was no significant correlation between the PI-HAT distance matrix and the Bray-Curtis distance (Mantel test, 10^5 permutations). As a supplementary test, using the same Mantel test, we computed correlation between the Bray-Curtis distance and the first ten principal components. The highest correlation was with PC3 with a correlation of 0.13 and p=0.027. However, not only is this correlation low, after correcting for multiple testing, the p value is above any reasonable cut-off.

Association testing was done using the same human genetic data against abundance levels of *Prevotella copri*, *Escherichia coli*, *Alistipes sp* CAG-435, *Faecalibacterium prausnitzii*, *Bacteroides vulgatus*, *Prevotella sp* TF12-30, *Prevotella sp* AM23-5, *Prevotella sp* AM42-24, *Bacteroides fragilis*, *Ruminococcaceae bacterium*. Again, because of the environmental difference between Bushbuckridge and Soweto we only used the 114 Bushbuckridge samples who passed QC both on human and microbiome data. Due to the small data size, we filtered out all SNPs with minor allele frequency less than 5%. Imputation was done using the Sanger imputation service using the African Genome Resource reference panel, yielding 7.757 million well imputed SNPs. None of the abundance levels had normal distributions, and in some cases there were extreme abundance levels far from the mean, which could bias the results given our small sample size. For this reason, we log-transformed the

abundance levels. We used the H3Agwas Association Testing pipeline (github.com/h3abionet/h3agwas) using GEMMA[8] as the underlying association testing tool. We used $5x10^{-8}$ as the *p*-value to account for multiple testing both with multiple SNPs and microbiome genera. The QQ-plots for all genera tested were well-behaved except for *Ruminococcaceae bacterium*, which we excluded from further analysis. Eight SNPs were below the cut-off, with several more in the suggestive level. This is shown in Table S6 which shows the genus, SNP, location of the SNP, the β-value and the p-value. Since we have a small sample and the *p*-values are not very far below the cut-off we present the results here as an initial analysis that might be useful for follow-up work.

The most interesting hit is in the *SLC2A10* gene, which encodes for a glucose transporter. Besides the two SNPs listed in the table there were several other SNPs in the same gene which had *p*-values above the cut-off but in a suggestive region. SNP rs10137347 is in a transcription factor binding site; the gene it is most associated with is *RNASE6* gene, which encodes a protein that is active in the urinary tract and which has antimicrobial properties, particularly against gram-negative bacteria (which Alistipes is). *FOXP1* is a gene with complex functions, but recent work shows that it is implicated in regulating the immune system. Finally, the GFRA1 protein is a neurotrophic factor, which is difficult to associate with the microbiome functioning.

Supplementary References

1. Clark, S. J. *et al.* Cardiometabolic disease risk and HIV status in rural South Africa: establishing a baseline. BMC Public Health **15**, 135 (2015).
2. Micklesfield, L. K. *et al.* Demographic, socio-economic and behavioural correlates of BMI in middle-aged black men and women from urban Johannesburg, South Africa. Glob. Health Action **11**, 1448250 (2018).
3. Pisa, P. T. *et al.* Nutrient patterns and their association with socio-demographic, lifestyle factors and obesity risk in rural South African adolescents. Nutrients **7**, 3464–3482 (2015).
4. Sedibe, M. H. *et al.* Dietary Habits and Eating Practices and Their Association with Overweight and Obesity in Rural and Urban Black South African Adolescents. Nutrients **10**, (2018).
5. Sedibe, H. M. *et al.* Qualitative study exploring healthy eating practices and physical activity among adolescent girls in rural South Africa. *BMC Pediatr.* **14**, 211 (2014).
6. Ramsay, M. *et al.* Regional and sex-specific variation in BMI distribution in four sub-Saharan African countries: The H3Africa AWI-Gen study. Glob. Health Action **11**, 1556561 (2018).
7. Purcell, S. *et al.* PLINK: a tool set for whole-genome association and population-based linkage analyses. Am. J. Hum. Genet. **81**, 559–575 (2007).
8. Zhou, X. & Stephens, M. Genome-wide efficient mixed-model analysis for association studies. Nat. Genet. **44**, 821–824 (2012).
