# Supplementary material for: Short- and long-read metagenomics of urban and rural South African gut microbiomes reveal a transitional composition and undescribed taxa
Source: Nat Commun. 2022 Feb 22;13:926. doi: 10.1038/s41467-021-27917-x (PMC8863827; doi:10.1038/s41467-021-27917-x)
Supplement: Supplementary file 3 — Description of Additional Supplementary Files [file 41467_2021_27917_MOESM3_ESM.docx]

File name: Supplementary Data 1

Description: Literature review of African gut microbiome studies

File name: Supplementary Data 2

Description: Sequence read counts before and after pre-processing

File name: Supplementary Data 3

Description: Relative abundance of microbial genera for each participant

File name: Supplementary Data 4

Description: Relative abundance of microbial species for each participant

File name: Supplementary Data 5

Description: Differentially abundant genera between Bushbuckridge and Soweto (positive log2 fold change indicates enrichment in Soweto samples)

File name: Supplementary Data 6

Description: HUMAnN MetaCyc pathway output for each participant, normalized to copies per million

File name: Supplementary Data 7

Description: Genome evaluation of medium- and high-quality short read metagenome-assembled genomes

File name: Supplementary Data 8

Description: Genome evaluation of medium- and high-quality nanopore long read metagenome-assembled genomes
