# Supplementary material for: Short- and long-read metagenomics of urban and rural South African gut microbiomes reveal a transitional composition and undescribed taxa
Source: Nat Commun. 2022 Feb 22;13:926. doi: 10.1038/s41467-021-27917-x (PMC8863827; doi:10.1038/s41467-021-27917-x)
Supplement: Supplementary file 12 — Reporting Summary [file 41467_2021_27917_MOESM12_ESM.pdf]

nature portfolio

Corresponding author(s): Ami S. Bhatt

Last updated by author(s): November 11, 2021

Reporting Summary

Nature Portfolio wishes to improve the reproducibility of the work that we publish. This form provides structure for consistency and transparency in reporting. For further information on Nature Portfolio policies, see our Editorial Policies and the Editorial Policy Checklist.

Statistics

For all statistical analyses, confirm that the following items are present in the figure legend, table legend, main text, or Methods section.

n/a	Confirmed	
[ ]	[x]	The exact sample size (*n*) for each experimental group/condition, given as a discrete number and unit of measurement
[ ]	[x]	A statement on whether measurements were taken from distinct samples or whether the same sample was measured repeatedly
[ ]	[x]	The statistical test(s) used AND whether they are one- or two-sided<br>*Only common tests should be described solely by name; describe more complex techniques in the Methods section.*
[ ]	[x]	A description of all covariates tested
[ ]	[x]	A description of any assumptions or corrections, such as tests of normality and adjustment for multiple comparisons
[ ]	[x]	A full description of the statistical parameters including central tendency (e.g. means) or other basic estimates (e.g. regression coefficient) AND variation (e.g. standard deviation) or associated estimates of uncertainty (e.g. confidence intervals)
[ ]	[x]	For null hypothesis testing, the test statistic (e.g. *F*, *t*, *r*) with confidence intervals, effect sizes, degrees of freedom and *P* value noted<br>*Give P values as exact values whenever suitable.*
[x]	[ ]	For Bayesian analysis, information on the choice of priors and Markov chain Monte Carlo settings
[x]	[ ]	For hierarchical and complex designs, identification of the appropriate level for tests and full reporting of outcomes
[x]	[ ]	Estimates of effect sizes (e.g. Cohen's *d*, Pearson's *r*), indicating how they were calculated

Our web collection on statistics for biologists contains articles on many of the points above.

Software and code

Policy information about availability of computer code

Data collection	No software was used for data collection
Data analysis	All R code for analysis and figure generation is available at https://github.com/bhattlab/SouthAfrica<br>All computational workflows for analysis can be found at https://github.com/bhattlab/bhattlab_workflows<br>Software used in this study: TrimGalore v0.6.5, SuperDeduper vl.2.0, BWA v0.7.17, Kraken v2.0.9, GTDB release 95, Bracken v2.2.0, sourmash v2.0.0, ShortBRED v0.9.3, PanPhlAn v3.1, SPAdes v3.15, Meta BAT v2.13 and v2.15, CONCOCT vl.1.0, MaxBin v2.2.7, DASTool vl.1.1, QUAST v5.0.2, CheckM v1.0.13, Prokka v1.14.6, Aragorn v1.2.38, Barrnap v0.9, dRep v3.2.0, Lathe v1, Flye v2.4.2, Pilon v1.23, Racon v1.4.10, minimap2 v2.17, Medaka v0.11.5, Pilon vl.23, Guppy v2.3.5, GTDBtk v1.4.1, VIBRANT v1.2.1, ResFams v1.2, MUSCLE v3.8.1551, FastTree v2.1.10, Fig Tree v1.4.4, R v4.0.2, MASS v7.3-53, ggsignif v0.6.0, ggpubr v0.4.0, vegan v2.5-6, cowplot v1.0.0, DESeq v1.28.0, genefilter v1.70.0, ggplot2 v3.3.2, ggrepel v0.8.2, gtools v3.8.2, harrietr v0.2.3, reshape2 v1.4.4, tidyverse v1.3.0, SkewIT v1, DNA-Plotter v18.1.0, HUMAaN v3.0.0, iTOL v6, stats v4.0.2, MaAsLin v2, khmer v3.0.0, MetaPhlAn v3

For manuscripts utilizing custom algorithms or software that are central to the research but not yet described in published literature, software must be made available to editors and reviewers. We strongly encourage code deposition in a community repository (e.g. GitHub). See the Nature Portfolio guidelines for submitting code & software for further information.

Data

Policy information about availability of data

All manuscripts must include a data availability statement. This statement should provide the following information, where applicable:

- Accession codes, unique identifiers, or web links for publicly available datasets
- A description of any restrictions on data availability
- For clinical datasets or third party data, please ensure that the statement adheres to our policy

All shotgun sequence data generated by this study, as well as metagenome-assembled genome sequences are deposited in the NCBI Sequence Read Archive under BioProject PRJNA678454. Participant-level metadata (age, BMI, blood pressure measurements, and concomitant medications) and human genetic data will be deposited in the European Genome-phenome Archive (EGA) under Study ID EGAS00001002482 and dataset ID EGAD00001006581.Comprehensive Antibiotic Resistance Database release 1.1.8 is available at https://card.mcmaster.ca/. Unified Human Gastrointestinal Genome collection data are available in the European Nucleotide Archive under study accession ERP116715. Genome Taxonomy Database release 95 is available at https://data.gtdb.ecogenomic.org/releases/.

Field-specific reporting

Please select the one below that is the best fit for your research. If you are not sure, read the appropriate sections before making your selection.

☒ Life sciences ☐ Behavioural & social sciences ☐ Ecological, evolutionary & environmental sciences

For a reference copy of the document with all sections, see nature.com/documents/nr-reporting-summary-flat.pdf

Life sciences study design

All studies must disclose on these points even when the disclosure is negative.

Sample size	Single samples were obtained from 190 adult women from two locations: Bushbuckridge Municipality (n=132) and Soweto (n=58). Sample size determination was based on participant availability and is comparable or exceeds sample sizes of other microbiome characterization studies.
Data exclusions	Samples from HIV+ individuals were excluded as a predetermined criterium as HIV+ status confounds microbiome composition comparisons. 118 samples from Bushbuckridge and 51 samples from Soweto were carried forward for further analysis.
Replication	As this study compares samples from two populations, we did not replicate the full study with an additional set of sample collection. We verified our classification-based analyses by classifying our data against various reference databases. All attempts at replication with various reference databases were successful. The analysis in this study is reproducible through the availability of our computational pipelines (see Software and Code availability).
Randomization	Participants/samples were not randomized into experimental groups for this study as we compared the microbiomes of two distinct communities. Samples were randomized across plates for DNA extraction and sequencing to avoid batch effects between groups. Covariates between groups were not controlled for, as the purpose of this study was to understand how geography and lifestyle covariates relate to microbiome composition.
Blinding	Investigators were not blinded to group during data collection. Blinding was not possible as participants were surveyed at their respective locales. Blinding was not relevant to our study as all data were processed through the same computational pipelines.

Reporting for specific materials, systems and methods

We require information from authors about some types of materials, experimental systems and methods used in many studies. Here, indicate whether each material, system or method listed is relevant to your study. If you are not sure if a list item applies to your research, read the appropriate section before selecting a response.

Materials & experimental systems

n/a	Involved in the study	
☒	☐	Antibodies
☒	☐	Eukaryotic cell lines
☒	☐	Palaeontology and archaeology
☒	☐	Animals and other organisms
☐	☒	Human research participants
☒	☐	Clinical data
☒	☐	Dual use research of concern

Methods

n/a	Involved in the study	
☒	☐	ChIP-seq
☒	☐	Flow cytometry
☒	☐	MRI-based neuroimaging

Human research participants

Policy information about studies involving human research participants

Population characteristics	All participants were women of ages 40-72 in Soweto and Bushbuckridge Municipality, South Africa. Population level covariates include increased population density and increased prevalence of piped water, electricity, and flush toilets in Soweto. Detailed population characteristics, including obesity statistics, medication summaries, and site characteristics are detailed in the manuscript.
Recruitment	Participants were recruited based on their prior participation in AWi-Gen (a cross-sectional study recruiting based on census data and random selection (Bushbuckridge) or recruiting based on participation in existing studies and geographical-based random selection (Soweto)). No self-selection bias is expected in these data. Participants were not compensated for participating.
Ethics oversight	Ethical oversight for human subject research by: Stanford IRB 43069, University of the Witswatersrand Human Research Ethics Committee M160121, Mpumalanga Provincial Health Research Committee MP 2017RP22_851.

Note that full information on the approval of the study protocol must also be provided in the manuscript.
