# Supplementary material for: Short- and long-read metagenomics of urban and rural South African gut microbiomes reveal a transitional composition and undescribed taxa
Source: Nat Commun. 2022 Feb 22;13:926. doi: 10.1038/s41467-021-27917-x (PMC8863827; doi:10.1038/s41467-021-27917-x)
Supplement: Supplementary file 13 — H3Africa AWI-Gen consortium [file 41467_2021_27917_MOESM13_ESM.docx]

**H3Africa Consortium AWI-Gen Study**

Core AWI-Gen group to be acknowledged in publications

**Historical Acknowledgement**

Investigators responsible for the conception and design of the AWI-Gen study include the following: Michele Ramsay (PI, Wits), Osman Sankoh (co-PI, INDEPTH), Stephen Tollman (Agincourt PI) and Kathleen Kahn (Agincourt), Marianne Alberts (Dikgale PI), Catherine Kyobutungi (Nairobi PI), Halidou Tinto (Nanoro PI), Abraham Oduro (Navrongo PI), Shane Norris (Soweto PI), and Scott Hazelhurst, Nigel Crowther, Himla Soodyall, and Zané Lombard (Wits).

The table below shows the members of the AWI-Gen Consortium who contributed significantly to the work of the consortium. Authors of specific papers are solely responsible for the papers to which their names are specifically attached; membership of the consortium does not in itself imply that the person takes responsibility for any paper.

**Affiliations**

1. Sydney Brenner Institute for Molecular Bioscience, University of the Witwatersrand, Johannesburg, South Africa
2. Division of Human Genetics, School of Pathology, Faculty of Health Sciences, University of the Witwatersrand, Johannesburg, South Africa
3. SAMRC/Wits Developmental Pathways for Health Research Unit, Faculty of Health Sciences, University of the Witwatersrand, Johannesburg, South Africa.
4. School of Human Development and Health, University of Southampton, Southampton, United Kingdom
5. SAMRC/Wits Rural Public Health and Health Transitions Research Unit (Agincourt), School of Public Health, Faculty of Health Sciences, University of the Witwatersrand, Johannesburg, South Africa
6. INDEPTH Network, East Legon, Accra, Ghana
7. School of Electrical & Information Engineering, University of the Witwatersrand, Johannesburg, South Africa
8. Navrongo Health Research Centre, Navrongo, Ghana
9. African Population and Health Research Centre, Nairobi, Kenya
10. Clinical Research Unit of Nanoro, Institut de Recherche en Sciences de la Santé, Nanoro, Burkina Faso
11. School of Health Care Sciences, Faculty of Health Sciences, University of Limpopo, Polokwane, South Africa.
12. School of Public Health, University of Ghana, Accra, Ghana.
13. Department of Chemical Pathology, National Health Laboratory Service, Faculty of Health Sciences, University of the Witwatersrand, Johannesburg, South Africa.

**Key AWI-Gen contributors over extended period**

| **Centre** | **Names** | **Affiliation** | **AWI-Gen 1** | **AWI-Gen 2** |
| --- | --- | --- | --- | --- |
| **Agincourt** | Stephen Tollman | 7, 8 | √ | √ |
|  | Alisha Wade | 7 | √ | √ |
|  | Chodziwadziwa Kabudula | 7 | √ | √ |
|  | Daniel Ohene-Kwofie | 7 | √ | √ |
|  | F. Xavier Gómez-Olivé | 7, 8 | √ | √ |
|  | Floidy Wafawanaka | 7 | √ | √ |
|  | Kathleen Kahn | 7, 8 | √ | √ |
|  | Mwawi Gondwe | 7 |  | √ |
|  | Rhian Twine | 7 | √ | √ |
|  | Ryan Wagner | 7, 8 | √ | √ |
| **APHRC** | Catherine Kyobutungi | 14 | √ | √ |
|  | Christopher Khayeka-Wandabwa | 14 | √ |  |
|  | Gershim Asiki | 14 | √ | √ |
|  | Isaac Kisiangani | 14 | √ | √ |
|  | Shukri Mohamed | 14 | √ | √ |
| **DIMAMO** | Marianne Alberts **§** | 16 | √ | √ |
|  | Solomon Choma | 16 |  | √ |
|  | Felistas Mashinya | 16 | √ |  |
|  | Given Mashaba | 16 |  | √ |
| **Nanoro** | Halidou Tinto | 15 | √ | √ |
|  | Herman Sorgho | 15 | √ | √ |
|  | Palwendé Romuald Boua | 15 | √ | √ |
| **Navrongo** | Abraham R Oduro | 13 | √ | √ |
|  | Godfred Agongo | 13 | √ | √ |
|  | Cornelius Debpuur | 13 | √ | √ |
|  | Engelbert Nonterah | 13 | √ | √ |
| **Soweto** | Shane A Norris | 5, 6 | √ | √ |
|  | Lisa Micklesfield | 5 | √ | √ |
|  | Vukosi Baloyi | 5 | √ | √ |
| **Wits/WHC** | Michèle Ramsay | 2, 4 | √ | √ |
|  | Ananyo Choudhury | 2 | √ | √ |
|  | Busisiwe Mthembu | 2 |  | √ |
|  | Cassandra Soo | 2 | √ | √ |
|  | Dhriti Sengupta | 2 | √ | √ |
|  | Ernest Tambo | 2 | √ |  |
|  | Francisco Camiña Ceballos | 2 | √ |  |
|  | Freedom Mukomana | 2 | √ | √ |
|  | Furahini Tluway | 2 |  | √ |
|  | Henry Wandera | 2 | √ | √ |
|  | Himla Soodyall | 4 | √ |  |
|  | Jean-Tristan Brandenburg | 2 | √ |  |
|  | Natalie Smyth | 2 | √ | √ |
|  | Nigel Crowther | 18 | √ | √ |
|  | Ovokeraye Oduaran | 2 | √ | √ |
|  | Scott Hazelhurst | 2, 9 | √ | √ |
|  | Stuart Ali | 2 | √ | √ |
|  | Theo Mathema | 2 | √ | √ |
|  | Tinashe Chikowore | 2 | √ |  |
|  | Yaniv Swiel | 2, 9 | √ | √ |
|  | Zané Lombard | 4 | √ |  |
| **INDEPTH** | Osman Sankoh | 8 | √ |  |
| **UG** | Pauline Tindana | 13, 17 | √ | √ |

§ Deceased
